# Supplementary material for: Fine-Resolution Mapping of TF Binding and Chromatin Interactions
Source: Cell Rep. 2018 Mar 6;22(10):2797–807. doi: 10.1016/j.celrep.2018.02.052 (PMC5863041; doi:10.1016/j.celrep.2018.02.052)
Supplement: Document S1. Supplemental Experimental Procedures and Figures S1–S5 [file mmc1.pdf]

Cell Reports, Volume *22*

Supplemental Information

Fine-Resolution Mapping of TF

Binding and Chromatin Interactions

Jenia Gutin, Ronen Sadeh, Nitzan Bodenheimer, Daphna Joseph-Strauss, Avital Klein-Brill, Adi Alajem, Oren Ram, and Nir Friedman

Supplementary Text: Detailed Protocol

Yeast growth

- Yeast cells were grown in YPD media at 30ºC with constant shaking to OD 0.6-0.8.

Yeast Cells fixation

- Add formaldehyde (1% final concentration) directly to cells and rotate 15 minutes at RT.
- Add glycine (0.125M final concentration from 2.5M stock) to the fixed cells and rotate 5 minutes at RT.
- Pellet cells by centrifugation (4000g, 5 minutes, 4ºC).
- Resuspend the cells pellet in ice cold ddH2O supplemented with a EDTA-free protease inhibitors cocktail (Roche).
- Pellet cells by centrifugation (4000g, 5 minutes, 4ºC).

Note: It is possible at this point to flash freeze the cell pellet and store at -80ºC.

Spheroplasting

- Resuspend the cell pellet in buffer Z (~ 5ul buffer Z per 1 OD600 of cells).
- Add zymolyase 20T (Seikagaku) at 0.15 - 0.5 units per 2x10^7 cells. Incubate cells at 30ºC for 20 minutes.
- To test spheroplasting efficiency, remove 1-5 µl of the cells into 1% SDS solution and check the cells under the microscope. Estimate spheroplasting efficacy (counted cells/expected # of cells*100). This number should be lower than 5%. If it is higher than 5% continue incubating the cells at 30ºC and test again.

MNase digestion (yeast)

Note: Since different TFs may exhibit distinct MNase sensitivity profiles, it is recommended to calibrate your MNase digest per specific TF by running a titration experiment. We find that 0.02-1 units of Worthington MNase per 10^7 cells to be a good range for initial titration. An alternative approach is to mix chromatin from different MNase digestion levels prior to immunoprecipitation.

- Pellet spheroplasts (6500 g, 10 minutes), remove the supernatant, and resuspend in NP buffer at final concentration of $0.2x10^7$ cells/µl.
- Prewarm samples to 37ºC for 5 minutes.
- Add MNase (Worthington) diluted in NP buffer for 20 minutes at 37ºC.
- Remove tubes to ice and add one volume of ice cold MNase stop buffer.

- Keep tubes on ice for 10-30 minutes (longer incubation increases yield), vortexed 3 x 10 seconds (this step increases chromatin yield but can be skipped when using 96 well plates to avoid sample spilling).
- Centrifuge samples (16,000g, 10 minutes, 4ºC - for 96 wells plates centrifuge 30 minutes at 5000g, 4ºC).
- Remove the supernatant containing the chromatin to fresh tubes or 96 well plate.

mESCs fixation and lysis: Cells were fixed with 1% formaldehyde for 15 minutes at room temperature, following by quenching with 0.125M glycine for 5 minutes at room temperature.

- Centrifuge cells, remove supernatant, and wash with ice cold PBS.
- Suspend cell pellet in 1 volume of ice cold PBS to get 10^7 cells/ml.
- Add equal volume of ice cold 2X cell lysis buffer
- Add the desired amount of MNase
- Mix well by pipetting up and down and incubate on ice for 10 minutes.
- Incubate 15 minutes at 37°C
- Stop MNase by adding 20mM EDTA.
- Incubate on ice for 30 minutes
- Centrifuged samples (16,000g, 10 minutes, 4ºC - for 96 wells plates centrifuge 30 minutes at 5000g, 4ºC).
- Remove the supernatant containing the chromatin to fresh tubes or 96 well plate.

MNase digest evaluation

- Remove 2-10µl of MNase digested chromatin to a fresh tube and adjust volume to 9ul with EB.
- Add 1µl of 0.5ug/µl RNase A and incubate for for 30 minutes at 37ºC.
- Add 40µl of proteinase K solution (10 mM Tris pH 8.0, 5 mM EDTA, 300 mM NaCl, 0.6% SDS) containing 50 units of proteinase K.
- Incubate for 2 hours at 37ºC, and for 12-16 hours at 65ºC.
- Isolate DNA by 2X SPRI beads cleanup, resuspend DNA in 20µl of 10mM Tris pH-8.0, and measure DNA concentration by Qubit.
- Expect total of ~ 10-100 ng of DNA
- Visualize DNA by TapeStation (Agilent) or agarose gel.

Chromatin immobilization

Note: The amount of DNA used for ChIP can vary and depends on the abundance of the target and antibody yield and specificity.

- Remove MNase digested chromatin samples to a fresh 96 well plate and adjust the volume to 80 µl with ice cold RIPA buffer and antibody (for specific details see antibodies section below).
- Incubate the samples with gentle tumbling for 2 hours to O/N at 4ºC.
- While samples are incubated wash protein G dynabeads three times in RIPA (yeast) or cell lysis buffer buffer (mESCs) (20µl beads per sample). Resuspend beads to the original volume with RIPA or cell lysis buffer.
- Centrifuge the samples shortly, add 20µl of protein G to each sample, and incubate the samples with gentle tumbling for an additional hour at 4ºC.
- Magnetize the samples and wash: 6 X RIPA buffer, 3 X RIPA 500 containing 500 mM NaCl), 3 X LiCl wash buffer. It is possible to use vacuum for these washes.
- **Important**: From this point on DO NOT use vacuum to aspirate the supernatant.
- Wash the beads 3 X 10 mM Tris pH 7.5 supplemented with protease inhibitors.

Chromatin barcoding

End repair:

- Resuspend the immobilized chromatin in 10µl of 10 mM Tris pH 7.5.
- Add 15 µl of end repair mixture and mix well by pipetting.
- Incubate for 22 minutes at 12°C followed by 22 minutes at 25°C.
- Magnetize beads and wash once in 150µl 10 mM Tris pH 8.0 and resuspend the beads in 20µl of 10 mM Tris pH 8.0.

A base addition:

- Add 10 µl of A-Base mix [10 mM Tris pH 8, 10 mM $MgCl_2$, 50 mM NaCl, 1 mM DTT, 0.58 mM dATP, 0.75 units Klenow fragment (NEB)] to the beads and mix well by pipetting.
- Incubate samples at 37°C for 30 minutes.
- Magnetize beads and wash once in 150µl 10 mM Tris pH 8.0 and resuspend the beads in 9µl of 10 mM Tris pH 8.0.

Adapters ligation:

- Add 2.5µl of indexed adapters (Blecher-Gonen et al., 2013) to each sample and mix well by pipetting. Add 17µl of ligation mix [14.5 µl of 2X quick ligase buffer (NEB), or Rapid ligation buffer (lucigen), and 2.5µl quick ligase (NEB) or NxGen T4 ligase], mix well by pipetting, and incubate at 25°C for 45 minutes.
- Magnetize beads and wash 3x 150µl RIPA

Note: Ligation of adaptors increases the size of DNA fragments bound by TFs and allow the isolation of fragments that are < 50bp (prior to adapter ligation) in subsequent steps.

Reverse crosslinks and DNA cleanup

- Resuspend the beads in 24μl of chromatin elution buffer supplemented with 1μl of 0.5μg/μl RNase A and incubate for for 30 minutes at 37ºC.
- Add 24μl of chromatin elution buffer supplemented with 1 μl of proteinase K (50 units/μl, epicenter).
- Incubate for 2 hours at 37ºC, and for 12-16 hours at 65ºC.
- At this point you can pool samples
- Isolate DNA by 2 X SPRI beads cleanup, resuspend DNA in 25μl of 10mM Tris pH-8.0.

Note: At this point it is possible to run a 4% agarose gel (E-Gel® EX Agarose Gels, 4%, Invitrogen) and gel purify DNA of desired size. This step can be skipped but it is recommended for elimination of adapter dimers and reduce background of nucleosomal DNA. We normally isolate DNA fragments of ~170-400bp which correspond to unligated fragments of 30-260bp.

Library amplification:

- Remove 23μl of the eluted chromatin into a fresh PCR tube. Add 2μl of barcoded amplification primers mix (Different barcode for each antibody used in second ChIP, see primer sequence below) and 25μl of 2 X Kapa hifi hotstart ready mix.
- Run PCR for 12-16 cycles.
  The number of PCR cycles depends on the ChIP yield. It is recommended to use the lowest number of cycles that yield sufficient library for next generation sequencing to reduce PCR duplicates.
- Isolate DNA by 0.8 X SPRI beads cleanup, resuspend in 20μl of 10mM Tris pH-8.0, and measure DNA concentration by Qubit.
  The total amount of amplified DNA can vary between 40-1000 ng of DNA.
- Visualize DNA by TapeStation. For example see Figure S1A.

Forward amplification primer:
5' - AATGATACGGCGACCACCGAGATCTACAC [8bp barcode] ACACTCTTTCCCTACACGAC
Reverse amplification primer:
5' - CAAGCAGAAGACGGCATACGAGAT

Buffers:

Buffer Z: 1 M sorbitol, 50 mM Tris 7.4, 10 mM β-mercaptoethanol (freshly added).

NP buffer: 10 mM Tris pH 7.4, 1 M sorbitol, 50 mM NaCl, 5 mM MgCl2, 1 mM CaCl2, and 0.075% NP-40, freshly supplemented with 1 mM β-mercaptoethanol, 500 μM spermidine, and EDTA-free protease inhibitor cocktail.

Cell lysis buffer: 50 mM Tris pH 8.0, 150 mM NaCl, 1% Triton X-100, 0.1% sodium deoxycholate, 5 mM CaCl2, EDTA-free protease inhibitor cocktail

MNase stop buffer: 220 mM NaCl, 0.2% SDS, 0.2% sodium deoxycholate, 10 mM EDTA, 2%,Triton X-100, EDTA-free protease inhibitor cocktail.

RIPA buffer: 10 mM Tris pH 8.0, 140 mM NaCl, 1 mM EDTA, 0.1% SDS, 0.1% sodium deoxycholate, 1% Triton X-100, EDTA-free protease inhibitor cocktail.

LiCl wash buffer: 10 mM Tris pH 8.0, 0.25 M LiCl, 0.5% NP-40, 0.5% Sodium Deoxycholate, 1 mM EDTA, EDTA-free protease inhibitor cocktail

End Repair mixture: 100 mM Tris pH 7.5, 20 mM MgCl2, 20 mM DTT, 2 mM ATP, 0.8 mM each dATP , dCTP, dGTP, dTTP, 12.5 units T4 polynucleotide kinase (NEB), 0.5 units T4 polymerase (NEB).

A-Base mix: 10 mM Tris pH 8, 10 mM MgCl2, 50 mM NaCl, 1 mM DTT, 0.58 mM dATP, 7.5 units Klenow fragment (NEB).

Chromatin elution buffer: 10 mM Tris pH 8.0, 5 mM EDTA, 300 mM NaCl, 0.6% SDS

Antibodies
The following antibodies were used in this study:

Antigen	Catlog #	µg antibody / ChIP
Anti Flag (M2)	Sigma	2
Anti CTCF	07-729 (Millipore)	2

Figure S1

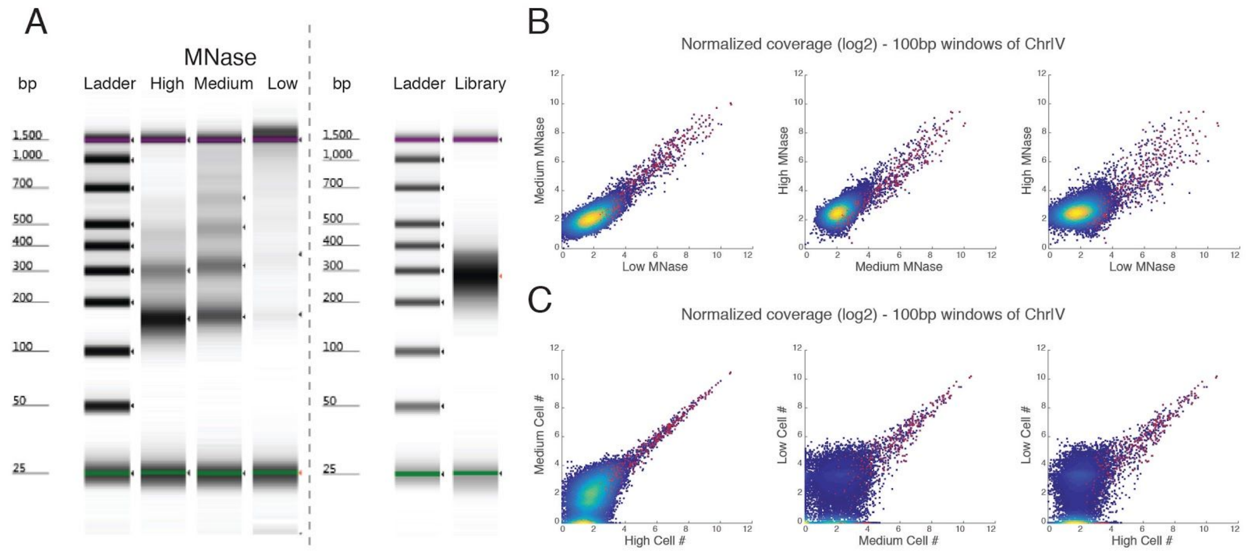

Figure S1: Comparison of MNase digestions and Input materials. Related to Figure 1B. **A.** Tape station chromatography of MNase digested DNA (left panel) or SLIM-ChIP library (right panel). Higher levels of MNase are characterized by larger proportion of mononucleosomes and shorter nucleosomal length. SLIM-ChIP library was isolated from gel as described in Methods. **B-C.** Scatter plots showing the sum of read coverage in 100bp windows across chrIV for different MNase concentrations and different cell numbers (as in Figure 1B). Locations of called peaks are highlighted.

Figure S2

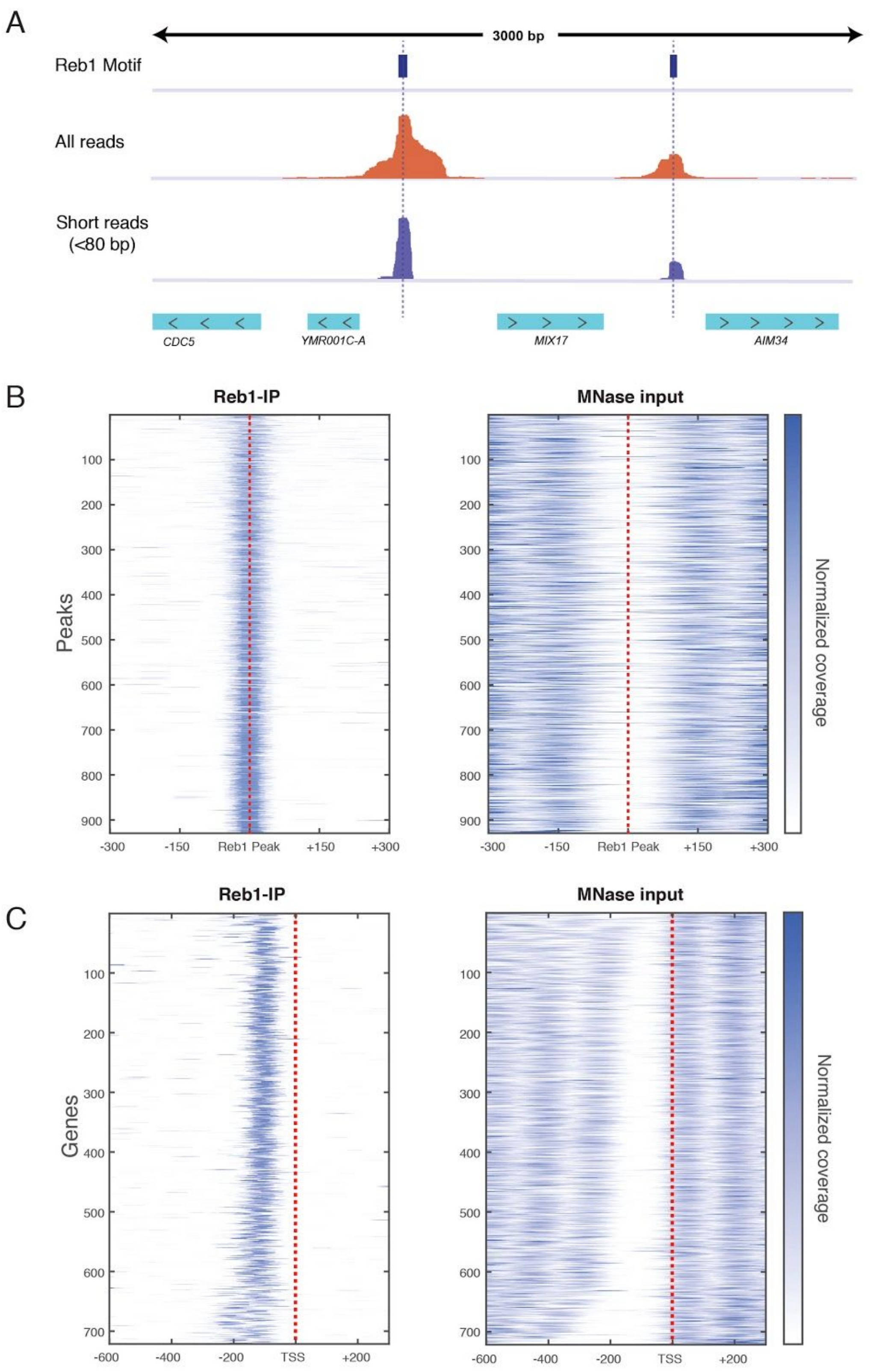
A
3000 bp
Reb1 Motif
All reads
Short reads
(<80 bp)
CDC5
YMR001C-A
MIX17
AIM34
B
Reb1-IP
MNase input
Peaks
100
200
300
400
500
600
700
800
900
-300
-150
Reb1 Peak
+150
+300
Normalized coverage
C
Reb1-IP
MNase input
Genes
100
200
300
400
500
600
700
-600
-400
-200
TSS
+200
Normalized coverage

Figure S2: Reb1 peak coverage. Related to Figures 1B-D. **A.** Genome browser view illustrating the signal from all reads versus short reads (<80bp). Focusing on short reads tightens the signal around the Reb1 binding motifs. **B.** Heat map of the read coverage of Reb1 ChIP signal (short reads) and MNase input signal(Weiner et al. 2015) centered on Reb1 peak (as in Figure 1C). **C.** Heat map of the same signals as B, aligned according to the TSS of genes with a Reb1 peak in their promoter.

Figure S3

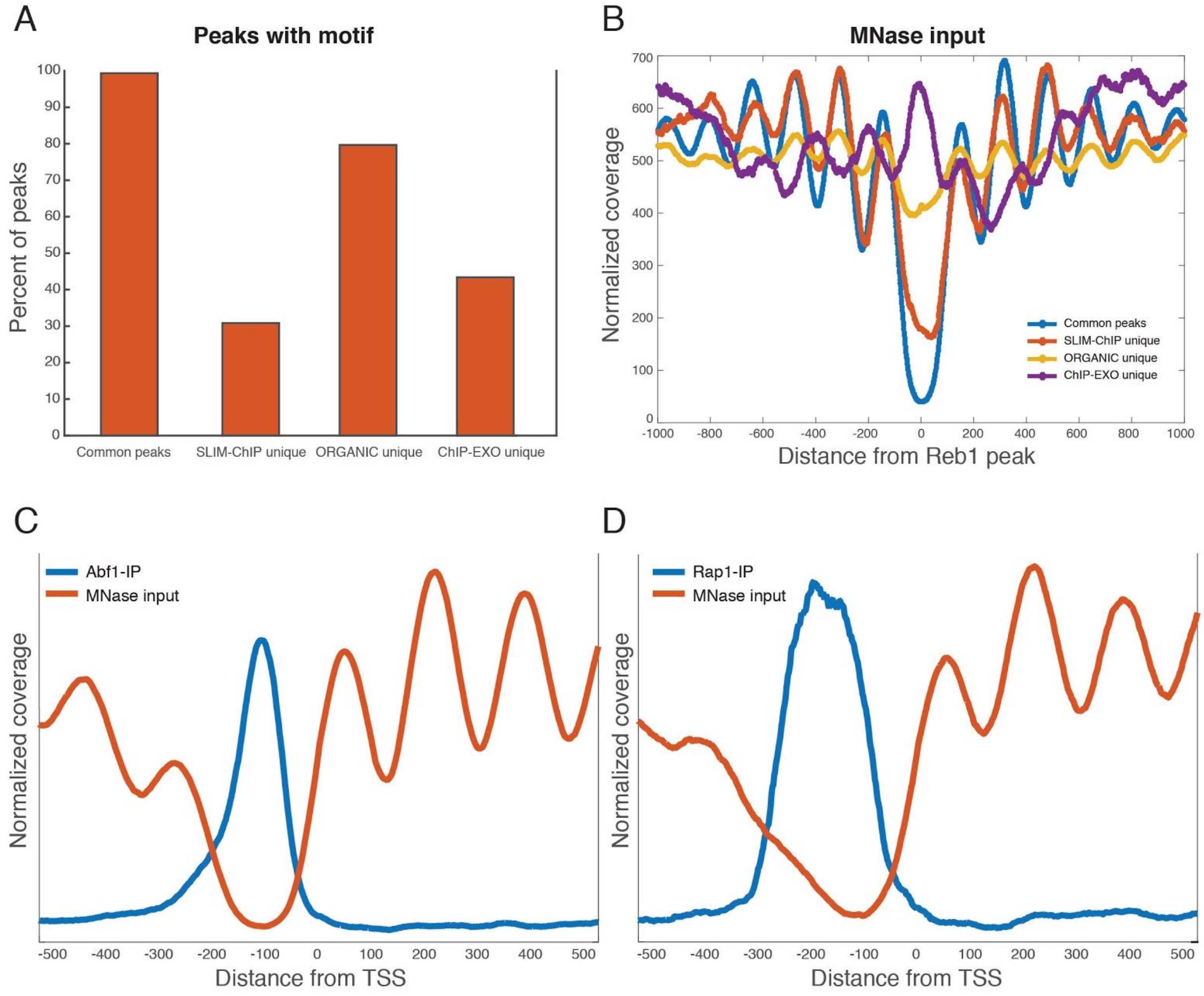

Figure S3: Reb1 across methods and profiling additional yeast transcription factors. **Related to Figure 2B and 3A.**. **A.** For selected groups in Figure 2B the percent of peaks with a motif is shown. The vast majority of common peaks contain a Reb1 binding motif. **B.** For selected groups in 2B the normalized coverage of MNase input (Weiner et al. 2015) signal around Reb1 peak centers is plotted. **C-D.** Normalized coverage of Abf1,Rap1 (short reads, 0-80bp) and MNase input signals around TSS. For each factor only promoters that contain a motif are used. MNase input track was adapted from Weiner et. al.

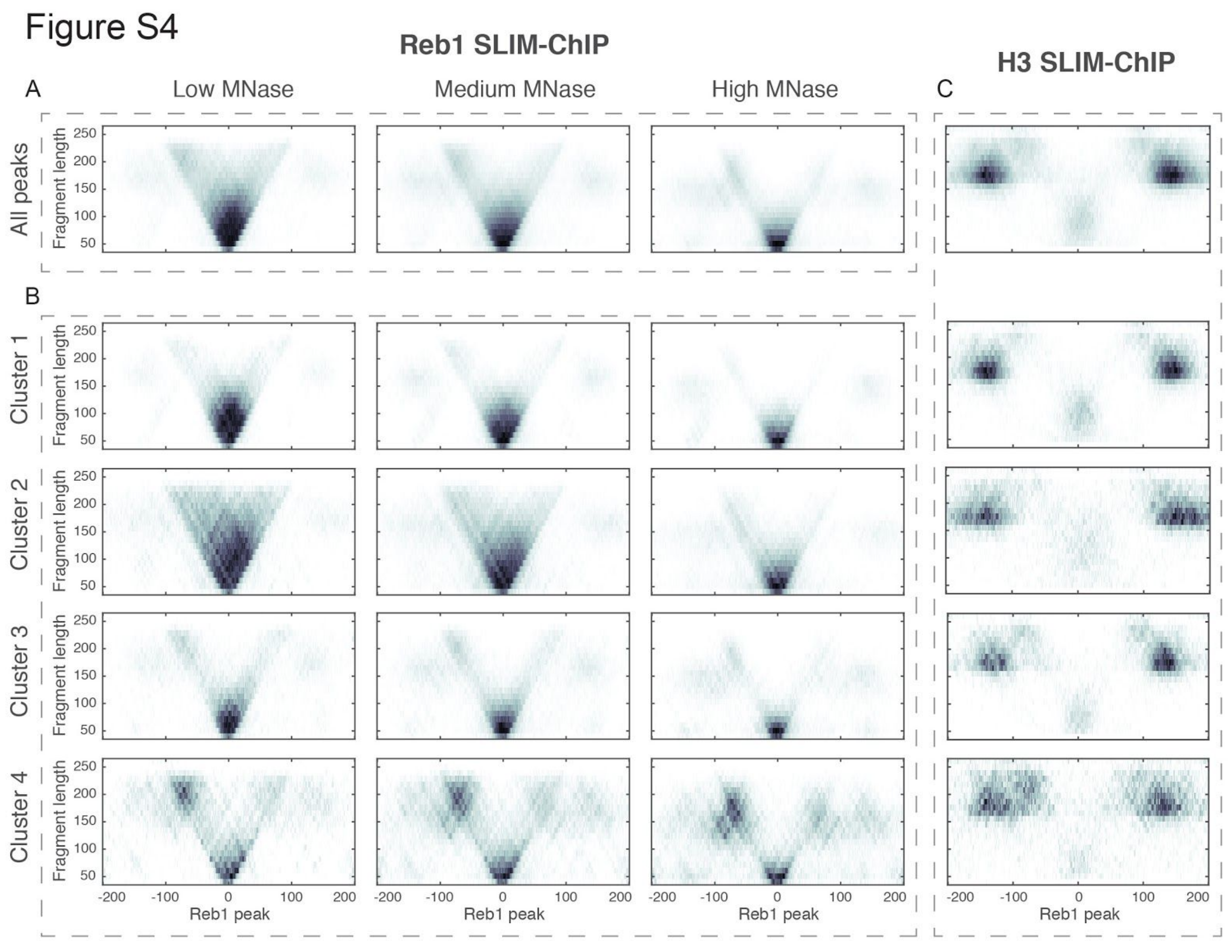

Figure S4: MNase sensitivity of read length patterns in clusters of Reb1 sites. Related to Figure 4. **A**. V-plots of all Reb1 sites in three MNase levels (as in Figure 4A). **B.** Same as A for each of the four clusters (as in Figure 4C). **C**. V-plots of H3 SLIM-ChIP for the groups shown in A and B.

Figure S5

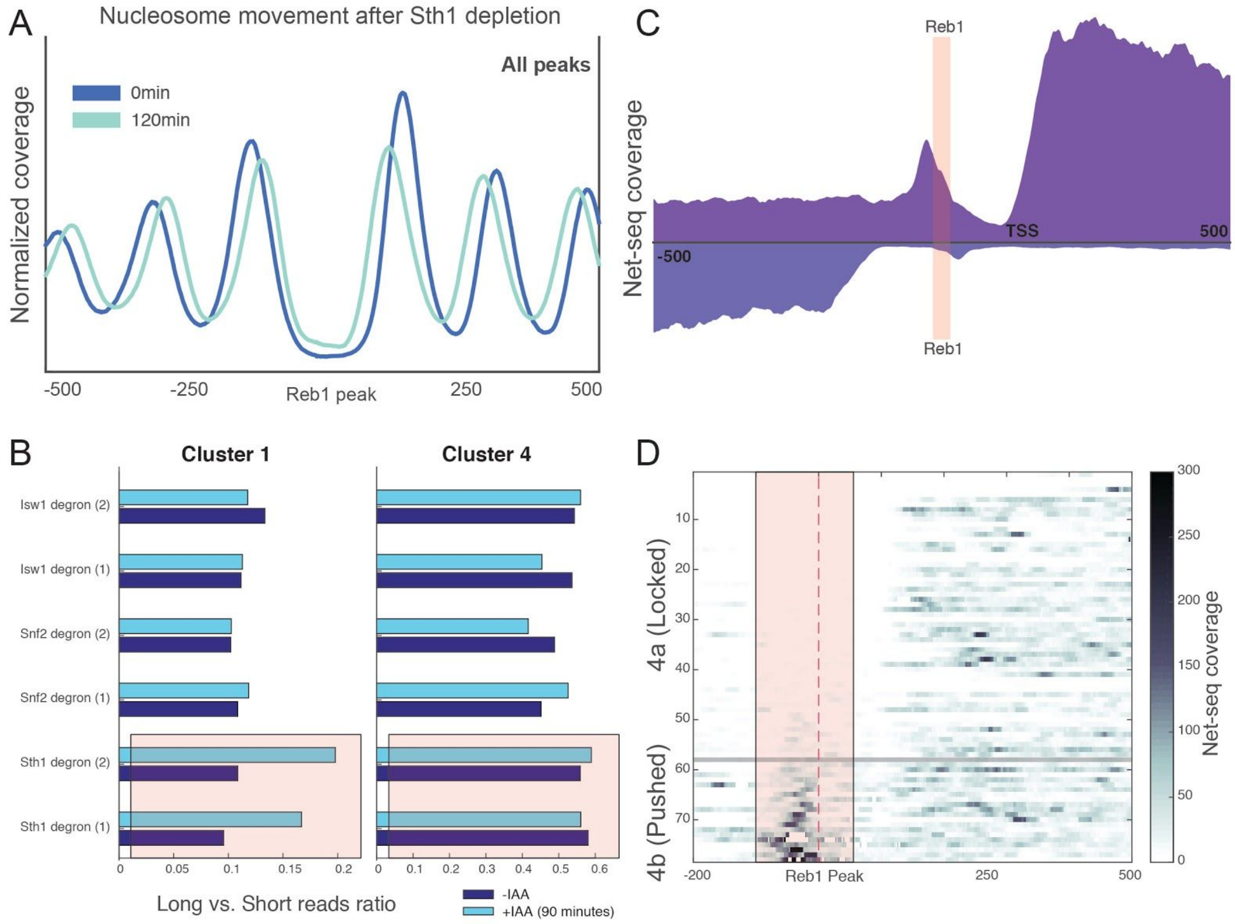

Figure S5: Effects of RSC depletion on Reb1 binding sites. Related to Figure 5. **A.** Nucleosome movement in Sth1 depletion for all Reb1 sites (as in Figure 5A,D). **B.** Examining change in fragment length in Reb1 SLIM-ChIP in three strains with depletion of a catalytic units of chromatin remodelers (Sth1 of RSC, Snf2 of SWI/SNF, Isw1 of ISW1a/b). Showing two repeats for each strain, comparing the ratio of total coverage of long fragment (>180bp) to the total coverage of short fragments (<110bp) counting over Reb1 sites in two clusters. Only in Sth1 depletion at Cluster 1 sites we observe a shift in the ratio. This shift corresponds to nucleosomes shifting to be adjacent to Reb1 when RSC is not active. **C.** Average NET-seq coverage at Reb1 binding sites (as in Figure 5B,C). **D.** Heat map of sense direction NET-seq coverage around each of the Reb1 sites in Cluster 4, oriented according to the nearest TSS. Rows (sites) are sorted according to total coverage in the highlighted region. We observe two subpopulations, one without transcription in the region and the other with.
