# Supplementary material for: Fine-Resolution Mapping of TF Binding and Chromatin Interactions
Source: Cell Rep. 2018 Mar 6;22(10):2797–807. doi: 10.1016/j.celrep.2018.02.052 (PMC5863041; doi:10.1016/j.celrep.2018.02.052)
Supplement: Document S2. Article plus Supplemental Information [file mmc2.pdf]

Cell Reports

Resource

Fine-Resolution Mapping of TF Binding and Chromatin Interactions

Graphical Abstract

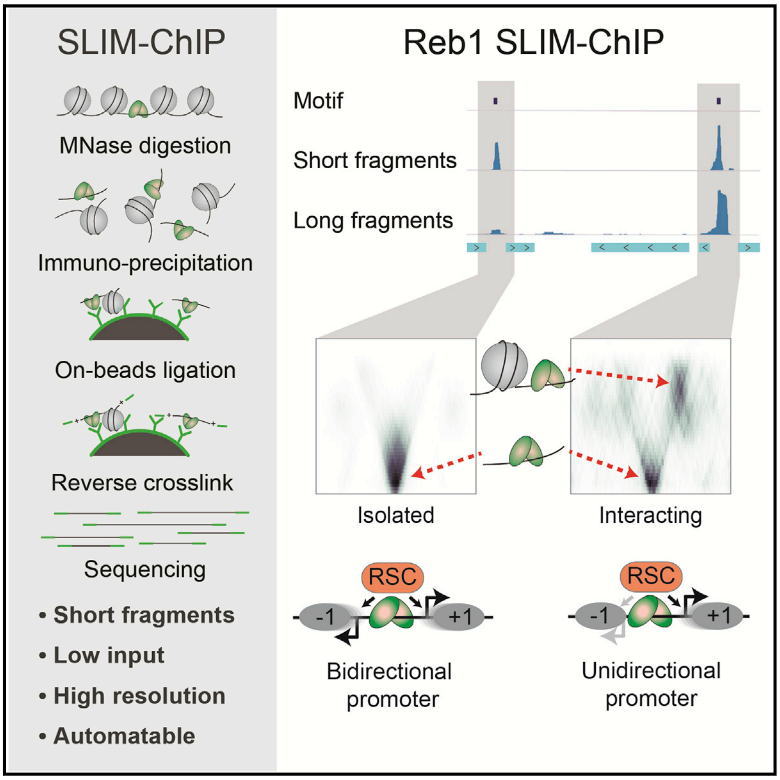

Authors

Jenia Gutin, Ronen Sadeh, Nitzan Bodenheimer, ..., Adi Alajem, Oren Ram, Nir Friedman

Correspondence

ronensadeh@gmail.com (R.S.), nir.friedman@mail.huji.ac.il (N.F.)

In Brief

Mapping transcription factors binding to DNA by chromatin immunoprecipitation sequencing is a key step in studying transcriptional programs. Gutin et al. introduce SLIM-ChIP, a simple, automation compatible protocol, that provides insights about the chromatin landscape at the bound sites. Using this protocol, they discover promoter architectures that enforce unidirectional transcription.

Highlights

- SLIM-ChIP is a low-input, robust, high-resolution, automatable TF mapping protocol
- SLIM-ChIP is applicable to a range of TFs from yeast to mammals
- DNA fragments from SLIM-ChIP provide targeted footprinting at the bound site
- RSC-mediated Reb1-nucleosome interactions affect promoter directionality

Data and Software Availability

GSE108948

Gutin et al., 2018, Cell Reports *22*, 2797–2807
March 6, 2018 © 2018 The Author(s).
https://doi.org/10.1016/j.celrep.2018.02.052

CellPress

Cell Reports
Resource

OPEN ACCESS
CellPress

Fine-Resolution Mapping of TF Binding and Chromatin Interactions

Jenia Gutin,[1,2,3] Ronen Sadeh,[1,2,3,*] Nitzan Bodenheimer,[1,2] Daphna Joseph-Strauss,[1,2] Avital Klein-Brill,[1,2] Adi Alajem,[2] Oren Ram,[2] and Nir Friedman[1,2,4,*]
[1]School of Computer Science and Engineering, The Hebrew University of Jerusalem, Jerusalem 9190401, Israel
[2]Institute of Life Sciences, The Hebrew University of Jerusalem, Jerusalem 9190401, Israel
[3]These authors contributed equally
[4]Lead Contact
*Correspondence: ronensadeh@gmail.com (R.S.), nir.friedman@mail.huji.ac.il (N.F.)
https://doi.org/10.1016/j.celrep.2018.02.052

SUMMARY

Transcription factor (TF) binding to DNA is crucial for transcriptional regulation. There are multiple methods for mapping such binding. These methods balance between input requirements, spatial resolution, and compatibility with high-throughput automation. Here, we describe SLIM-ChIP (short-fragment-enriched, low-input, indexed MNase ChIP), which combines enzymatic fragmentation of chromatin and on-bead indexing to address these desiderata. SLIM-ChIP reproduces a high-resolution binding map of yeast Reb1 comparable with existing methods, yet with less input material and full compatibility with high-throughput procedures. We demonstrate the robustness and flexibility of SLIM-ChIP by probing additional factors in yeast and mouse. Finally, we show that SLIM-ChIP provides information on the chromatin landscape surrounding the bound transcription factor. We identify a class of Reb1 sites where the proximal −1 nucleosome tightly interacts with Reb1 and maintains unidirectional transcription. SLIM-ChIP is an attractive solution for mapping DNA binding proteins and charting the surrounding chromatin occupancy landscape at a single-cell level.

INTRODUCTION

Binding of transcription factors (TFs) to specific DNA sequences is fundamental for regulation of transcription and chromatin structure. Our understanding of transcription factor binding *in vivo* is based on mapping their occupancy along the genome, mainly by chromatin immunoprecipitation (ChIP) (Solomon et al., 1988). Coupling of ChIP to next-generation sequencing technology allows genome-wide mapping of transcription factors in a single experiment (Mikkelsen et al., 2007). Typical ChIP protocols involve cross-linking of proteins to DNA prior to DNA shearing by sonication, immunoprecipitation with an antibody against the transcription factor of interest, release of bound DNA, and next-generation sequencing compatible library preparation. Drawbacks of standard ChIP-sequencing (ChIP-seq) assays are the requirement for large amounts of sample material and the relatively low resolution (~200–500 bp) due to the size of DNA fragments generated by chromatin sonication. Moreover, most ChIP assays are not readily compatible with high-throughput practices, which limits the number of samples that can be processed simultaneously.

In recent years, several improvements have been developed to overcome these limitations. The amount of initial material required can be dramatically reduced by ligation of barcoded adapters directly to chromatin fragments prior to isolation of DNA (van Galen et al., 2016; Lara-Astiaso et al., 2014). Barcoding allows pooling of many samples before library amplification by PCR. Dramatically increased resolution has been achieved by the ChIP-exo (Rhee and Pugh, 2011) and ORGANIC (Kasinathan et al., 2014) protocols. In ChIP-exo cross-linked chromatin is treated with exonuclease following the immunoprecipitation step to allow precise mapping of transcription factor-DNA cross-linking sites. In ORGANIC this is achieved by micrococcal nuclease (MNase) digestion of native chromatin. However, ChIP-exo and ORGANIC require a large number of cells (>10^9 in the original protocols) and involve several additional biochemical and/or molecular steps compared to conventional ChIP-seq. One obstacle in adapting ChIP protocols to high-throughput workflow is chromatin shearing by sonication. This obstacle can potentially be bypassed by specialized sonicators (Garber et al., 2012), yet such instruments are not commonly available. Alternatively, post-lysis (Albert et al., 2007; Kasinathan et al., 2014; Liu et al., 2005; Skene and Henikoff, 2015; Tsankov et al., 2015; Weiner et al., 2015) or *in situ* (Zentner et al., 2015) enzymatic digestion (e.g., MNase) do not require specialized equipment. Despite significant advancements in transcription factor mapping, currently available protocols still suffer from at least one of the limitations mentioned above.

Here, we combine the benefits of on-beads barcoding and MNase digestion, developing small-fragment-enriched, low-input, indexed MNase ChIP (SLIM-ChIP) (Figure 1A). We use MNase digestion rather than sonication, which allows reproducible chromatin fragmentation in small volumes using only liquid handling steps. Additionally, because MNase has both endo- and exo-nuclease activity, MNase digestion generates DNA fragments which are nearly the size of the DNA protected by the transcription factor and as a result can potentially provide high-resolution mapping of transcription factor binding sites

 © 2018 The Author(s).
This is an open access article under the CC BY license (http://creativecommons.org/licenses/by/4.0/).

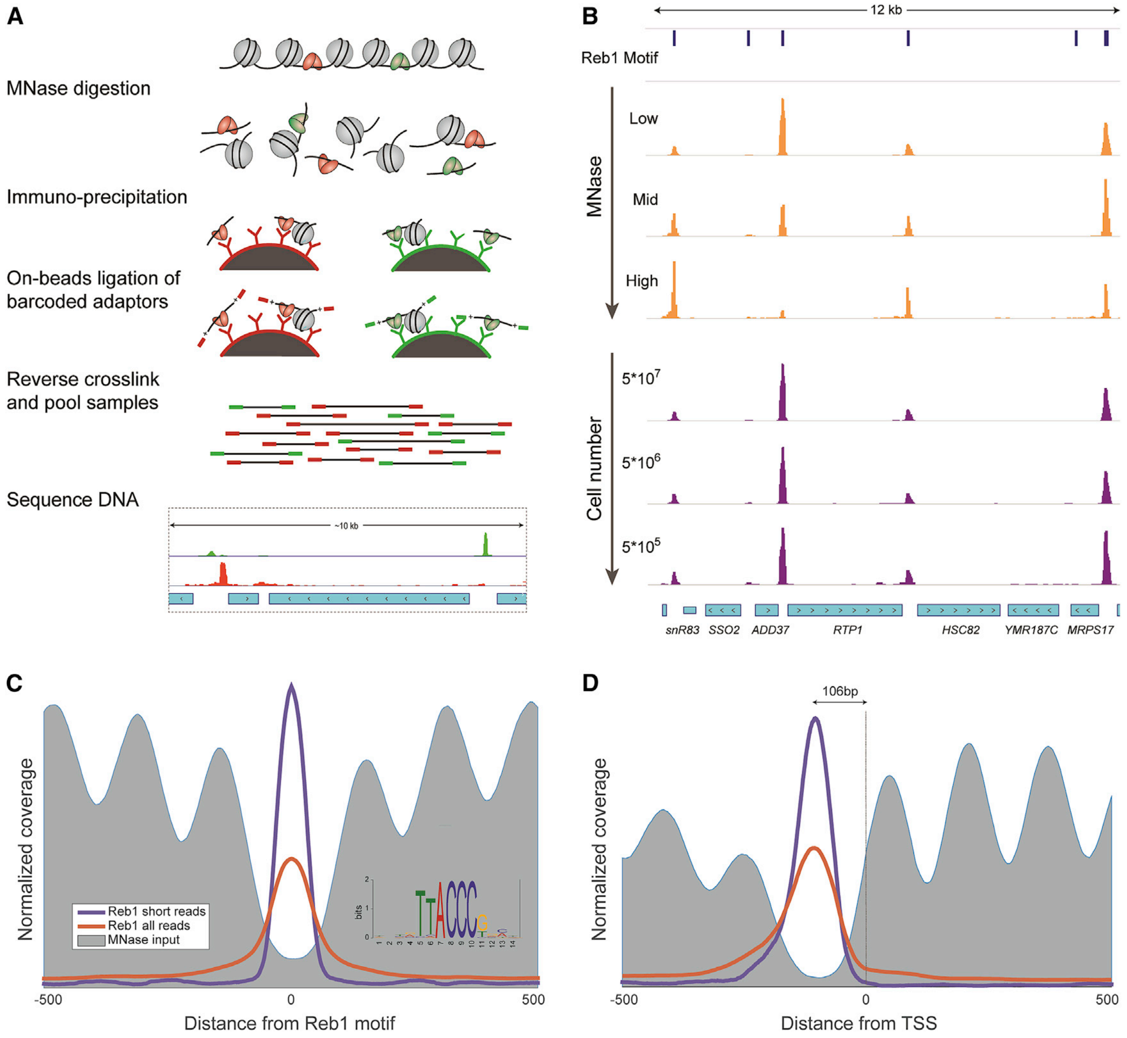

Figure 1. SLIM-ChIP Is Robust to MNase and Input Levels

(A) Outline of the SLIM-ChIP method. MNase-digested cross-linked chromatin is barcoded on beads allowing for multiplexing and efficient capturing of small transcription factor-bound DNA fragments.

(B) Genome browser view of Reb1 binding across range of MNase digestion (1X,9X,27X) and input material levels. The top track (Reb1 motif) marks consensus Reb1-binding motifs.

(C and D) Reb1-binding peaks are depleted for nucleosomes (C) and positioned at a typical distance from the TSS (D). The MNase input track was adapted from Weiner et al. (2015). Fragments of less than 80 bp were considered short reads. The Reb1 logo was generated for peaks called using short-reads data.

See also Figures S1 and S2.

(Henikoff et al., 2011). Finally, we utilize on-bead chromatin indexing workflow (Lara-Astiaso et al., 2014) in which immobilized chromatin is indexed prior to cross-linking reversal. The ligation of DNA adapters to chromatin prior to DNA purification results in an increase in DNA fragment size, which allows purification of otherwise too short fragments by conventional and high-throughput-compatible methods. As we show, SLIM-ChIP provides a simple, robust, low-input, and high-resolution transcription factor mapping method compatible with automation.

The SLIM-ChIP protocol starts with MNase digestion of chromatin (Figure 1A; Experimental Procedures). After stopping the MNase reaction by chelating calcium, the chromatin fragments are incubated with antibodies. The antibody-chromatin complexes are then immobilized to magnetic beads. These immobilized chromatin fragments are indexed by adaptor ligation using a slightly modified iChIP protocol (Lara-Astiaso et al., 2014; Sadeh et al., 2016). Subsequently, the chromatin fragments are reverse-crosslinked and DNA is purified. At this stage the samples can be pooled (from different starting cell populations, or from ChIP with various antibodies), as each sample is identified by a unique index sequence. The indexed DNA fragments are amplified by PCR. The resulting library is paired-end sequenced to reconstruct original fragments. These sequenced fragments are then de-multiplexed using read indexes.

RESULTS

SLIM-ChIP Reconstructs Rules of REB1 Binding

As a test case, we performed SLIM-ChIP to map Reb1 binding in *Saccharomyces cerevisiae* (Figure 1B). Reb1 is an essential yeast DNA-binding protein whose binding is seen as a priming event for the binding of other DNA-binding proteins. In particular, Reb1 has been implicated in establishing chromatin organization by steric inhibition of nucleosomes and preventing inappropriate RNA polymerase II read-through at transcription termination sites (Angermayr et al., 2003; Colin et al., 2014). As such, it is considered a stably bound factor. Previous studies mapped several hundred Reb1-binding locations across the yeast genome, residing primarily in nucleosome-depleted regions (NDRs) upstream of transcription start sites and downstream of transcription termination sites (Kasinathan et al., 2014; Rhee and Pugh, 2011).

We asked whether the Reb1 peaks we recover by SLIM-ChIP recapitulate known hallmarks of Reb1 binding preferences. First, we searched for sequence motifs enriched at SLIM-ChIP Reb1 peak centers (Experimental Procedures) recovering the consensus sequence motif (Figure 1C) that has been previously reported for Reb1 both *in vivo* and *in vitro* (Kasinathan et al., 2014; Liaw and Brandl, 1994; Rhee and Pugh, 2011). Second, because Reb1 is known to localize to NDRs *in vivo*, we aligned Reb1 peaks and compared them to MNase input signal. Consistent with prior studies, we find that Reb1 peaks are highly enriched in NDRs. Limiting our analysis to shorter fragments, which closely delimit binding locations, further highlights this pattern (Figures 1C, S2A, and S2B). Third, Reb1 binds upstream of transcription start sites (TSSs). Aligning promoters according to TSS shows that Reb1 peaks have a specific distance preference upstream of the TSS (Figures 1D and S2C).

SLIM-ChIP Robustly Detects Transcription Factor Binding Events

To test the robustness of SLIM-ChIP we applied it to chromatin from mid-log growing yeast cells digested with different concentrations of MNase (Figure 1B). The degree of MNase digestion may affect the size distribution of the chromatin fragments. Moreover, some regions are more susceptible to digestion than others and thus might be differentially represented at various digestion levels (Henikoff et al., 2011; Weiner et al., 2010). Overall, we find that the genomic loci bound by Reb1 are robustly captured across a range of different MNase levels (Figures 1B, S1A, and S1B), although there was some variability in local occupancy at Reb1-binding regions reflecting local susceptibility to MNase digestion (Figure 1B). This concern can be circumvented by combining chromatin preparations digested to different degrees.

SLIM-ChIP Is Compatible with Low Input

In many ChIP experiments, the amount of biological material needed to achieve a reliable signal can be a limiting factor. Titrating the number of input cells in Reb1 SLIM-ChIP over two orders of magnitude (5×10^7 to 5×10^5 cells) does not alter the signal (Figures 1B and S1C). This dramatic reduction in the amount of input cells enables the entire procedure, including cells growth and treatment, to be carried out in a standard 96-well plate. Therefore, SLIM-ChIP facilitates mapping of transcription factors in systematic high-throughput studies across many mutant backgrounds or growth conditions. Alternatively, the reduced amount of input enables using this method on rare subpopulations of cells such as those obtained by biopsies or fluorescence-activated cell sorting (FACS) from larger heterogeneous cell populations.

SLIM-ChIP Reconstructs the Reb1 Binding Map

ChIP of Reb1 has been used previously as a benchmark for several methods studying transcription factor binding. As such, it enables detailed comparison of multiple methods in the same biological system. Here, we compare SLIM-ChIP with two recent methods: ChIP-exo, in which exonuclease digestion of DNA up to the cross-linked nucleotide provides state-of-the-art spatial resolution (Rhee and Pugh, 2011), and ORGANIC, a method that uses MNase digestion in combination with immunoprecipitation of native chromatin (without cross-linking) (Kasinathan et al., 2014). In both cases, we compared our data to the list of predicted binding peaks, as defined by the respective authors, as well as to the raw coverage counts.

Examining the peaks identified by the three methods along the genome, we observe strong general agreement (Figure 2A as a representative region). Systematically comparing peak locations (Figure 2B; Experimental Procedures) reveals a large number of core peaks (743) detected by all three methods. These core peaks are enriched in intergenic regions (Figure 2C), contain Reb1 motif (Figure S3A), are nucleosome-depleted (Figure S3B), and have high sequence coverage in all three methods (Figures 2D–2F).

One exception is a large group of peaks (954) that are unique to ORGANIC. These peaks are different than the core peaks in three main aspects. First, more than 50% of ORGANIC-unique peaks are located within open reading frames (ORFs) compared to ~10% of core peaks (Figure 2C), Second, they reside in nucleosome-occupied locations (Figure S3B). Third, ORGANIC-unique peaks have significantly lower coverage (an average normalized coverage of 13 versus 286 reads per peak; Figure 2F). However, ORGANIC-unique peaks are enriched with Reb1 motif (Figure S3A). These observations are consistent with technical issues such as binding of soluble Reb1 to cryptic loci containing a Reb1 motif during the native ChIP procedure (which does not include a cross-linking step); alternatively, it is possible that higher sequencing depth captures peaks representing rare subpopulations of cells in which Reb1 is bound in a region that is only transiently accessible to it.

Together, these observations demonstrate that all three methods identify a large set of core peaks, which most likely represent sites that are highly occupied by Reb1 in a mid-log yeast population. We do observe additional peaks outside this core set. These peaks tend to have lower read coverage and thus may correspond to weaker or variable binding of Reb1 or alternatively can be due to technical biases and noise (Teytelman et al., 2013).

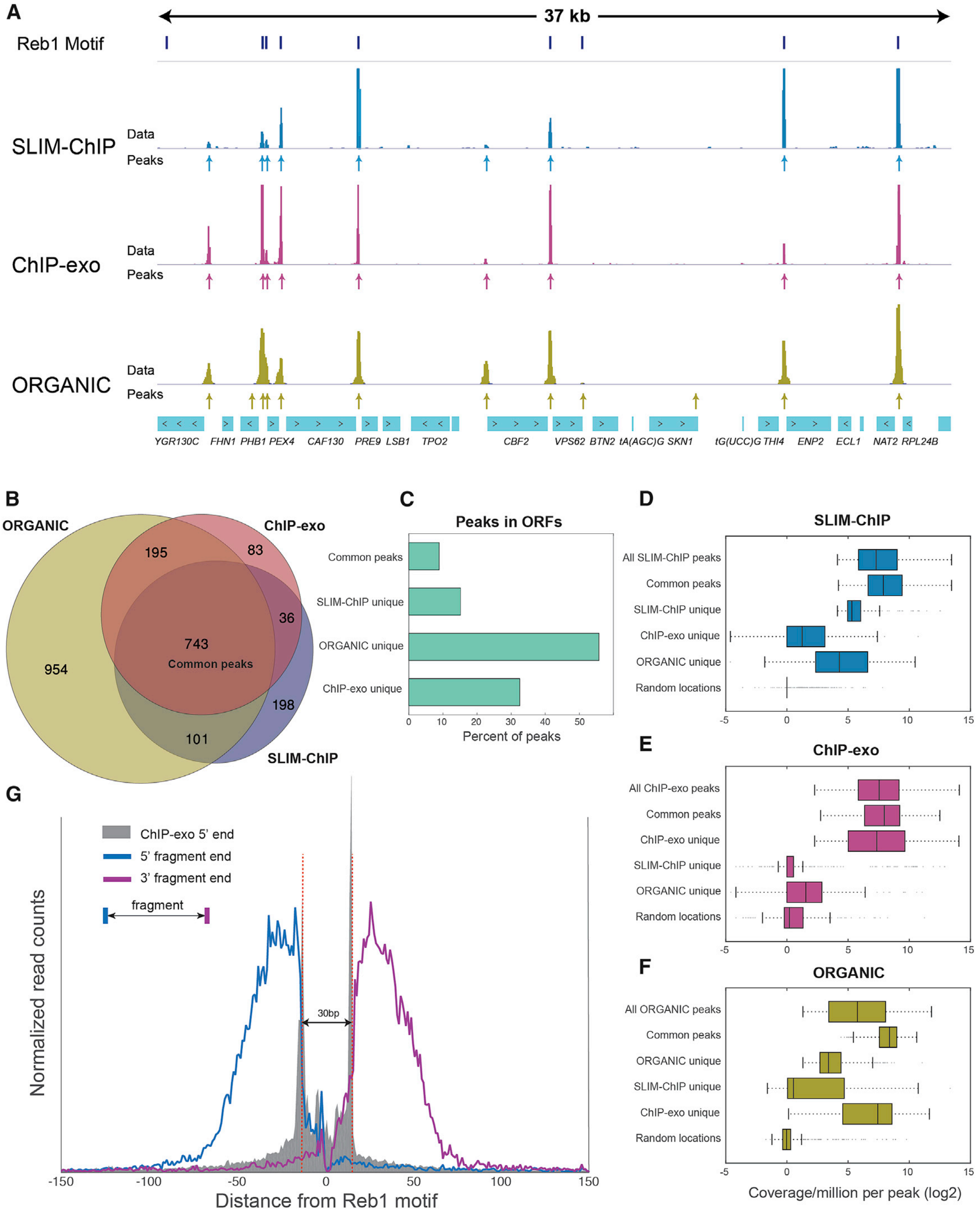

Figure 2. SLIM-ChIP Precisely Reconstructs the Reb1 Genome-wide Binding Profile

(A) Genome browser view of the Reb1-binding signal generated by SLIM-ChIP, ChIP-exo (Rhee and Pugh, 2011), and ORGANIC (Kasinathan et al., 2014). The data track shows coverage along the genome, and the peak tracks (arrows) show peak locations as provided by the respective authors.

(B) Venn diagram view of the overlap between Reb1 peaks as determined by the different methods. See also Figure S3.

(C) For selected groups in (B), the percentage of peaks with a center within an ORF.

(D–F) Boxplots showing the distribution of the normalized read coverage per peak in different peak groups for SLIM-ChIP (D), ChIP-exo (E), and ORGANIC (F). For each of the reported peaks, we calculated the sum of read coverage within the peak (±75 bp from peak summit). As a background, 500 random genomic locations were sampled.

(G) Reb1 protects ~30 bp around its core motif. All motif-containing peaks were oriented in the same direction relative to their Reb1 motif. The 5′ (blue) and 3′ (purple) ends of the reads were stacked to show the distinct protection pattern in the 30 bp around the motif.

SLIM-ChIP Resolution Is Comparable with ChIP-Exo

Our current understanding is that MNase has preference to endonucleolytically cleave protein-free DNA and further digest the cleaved fragments until it encounters shielded DNA or a formaldehyde cross-linking point. Thus, we reasoned that many of our ChIP fragments should end at such sites. Indeed, when plotting the location of fragment ends flanking the Reb1 peaks, we see sharp boundaries in a 30-bp window around the motif (Figure 2G). These boundaries match the exo-nuclease fragment ends reported by ChIP-exo (Figure 2G, gray outline). Interestingly, in ChIP-exo, all fragments are digested up to the cross-linking points, while in SLIM-ChIP, fragments ends are determined by the protection of protein-bound DNA and cross-linked residues from MNase digestion. We find this information highly useful for probing the transcription factor's local chromatin architecture beyond the cross-linking point, which does not exactly reflect the local protection pattern (see below).

Profiling Additional Yeast Transcription Factors

Having validated our method for Reb1, we next applied SLIM-ChIP to probe the binding pattern of Rap1 and Abf1, two additional well-studied yeast transcriptional regulators. As observed for Reb1, the binding patterns of Abf1 and Rap1 recover the expected landscape, with pronounced peaks (Figure 3A). Motif search for these peaks recovers the known binding preferences of both factors (Harbison et al., 2004). Consistent with previous reports, we observe Abf1 binding in NDRs upstream of genes, where it plays a role similar to that of Reb1. Similarly, we observe Rap1 binding in promoters of ribosomal genes, which have wider NDRs. Both transcription factors have little overlap with MNase nucleosome mapping (Figures S3C and S3D).

SLIM-ChIP Is Compatible with Mammalian Cells

Our method should in principle be readily applicable to a wide range of organisms. As a concrete example, we used it to map the binding of CTCF, a key chromatin organizer, in mouse embryonic stem cells (ESCs). The resulting binding map recapitulates earlier mapping of CTCF in the same cells (Figure 3B). Again, we examined the robustness to MNase concentration spanning a 64-fold difference. As expected, higher levels of MNase digestion resulted in tighter peaks around binding locations and lower number of reads, as MNase digests more of the protected DNA (Figure 3C).

The MNase digestion pattern around CTCF peaks uncovers a complex protection pattern, which is compatible with multiple zinc-finger DNA-binding domains in CTCF (Nakahashi et al., 2013) (Figure 3D). This pattern is the result of the protection provided by CTCF, preferred cross-linking sites, and MNase digestion preference. While further analysis may shed light on specific features of CTCF binding to DNA, it is clear that fragment ends tightly cluster around the CTCF binding consensus, attesting to the high resolution provided by SLIM-ChIP.

Fragment Length Reports on Transcription Factor-Nucleosome Interactions

An important challenge in the chromatin field is the reconstruction of the binding configurations of chromatin factors and their interactions (e.g., whether two factors compete for mutually exclusive binding or co-bind together). This problem can be tackled by methods for DNA accessibility footprinting (Buenrostro et al., 2013; Henikoff et al., 2011; Kent et al., 2011; Vierstra et al., 2014); however, the interpretation of co-occurrence from such data are indirect and requires prior knowledge of the factors' binding patterns. Combinatorial ChIP, where two or more factors are sequentially immunoprecipitated (Sadeh et al., 2016; Weiner et al., 2016), provides direct evidence of the interaction; however, it can be experimentally challenging and detects only predetermined pairs of interactions. Another strategy focused specifically on the interactions of a factor with nucleosomes by performing ChIP pull-down for the factor and then purifying and sequencing only the mononucleosome-sized DNA fragments (Koerber et al., 2009). This strategy detects interaction of nucleosome-bound factors. However, since it focuses on a fixed range of fragment lengths and does not compare to the distribution of shorter and longer fractions, it only collects partial information about the configuration of the co-binding patterns of the factor and the nucleosome.

We reasoned that SLIM-ChIP provides an alternative approach to focus on interactions of a target transcription factor. SLIM-ChIP captures DNA fragments enriched for the bound transcription factor. The boundaries of these fragments are largely determined by the protection pattern of the DNA as in MNase footprinting (Henikoff et al., 2011; Kent et al., 2011). Thus SLIM-ChIP provides a targeted footprinting approach. We therefore surmised that this type of information can reveal the binding and interaction landscape around transcription factor binding sites at a single-cell resolution.

We first analyzed fragment size distribution around Reb1 peaks oriented according to the TSS of the closest gene (Figure 4A). As expected, most fragments are short and centered around Reb1 motif. However, we also detect a significant subpopulation of 160- to 220-bp fragments, compatible with Reb1 interacting (co-bound) with a single nucleosome. These long fragments are asymmetrically distributed in relation to the TSS, suggesting that a subpopulation of Reb1 might preferentially interact with the −1 nucleosome (Figure 4A). These long fragments are relatively insensitive to MNase levels (Figure S4A), suggesting that they likely represent a stable interaction of Reb1 with the −1 nucleosome at a subpopulation of Reb1-bound promoters. This is in agreement with previous results (Koerber et al., 2009). We also detect mid range fragments (~150 bp) that are centered on Reb1 sites; however, these fragments almost completely disappear at high MNase levels (Figure S4A), suggesting that they likely represent long relatively unprotected DNA surrounding Reb1 sites.

Reb1 Footprint Defines Promoter Subtypes

We next examined the fragment length distribution at the level of single Reb1-binding sites (Figure 4B). Clearly, some binding sites are highly enriched for long fragments (Figure 4B, right gray box), while others are depleted (Figure 4B, left gray box). This suggests that promoters exist in several Reb1-related configurations based on their interaction with the proximal −1 nucleosome. To further explore the possible configurations, we clustered Reb1 sites based on their fragment lengths profile. Since

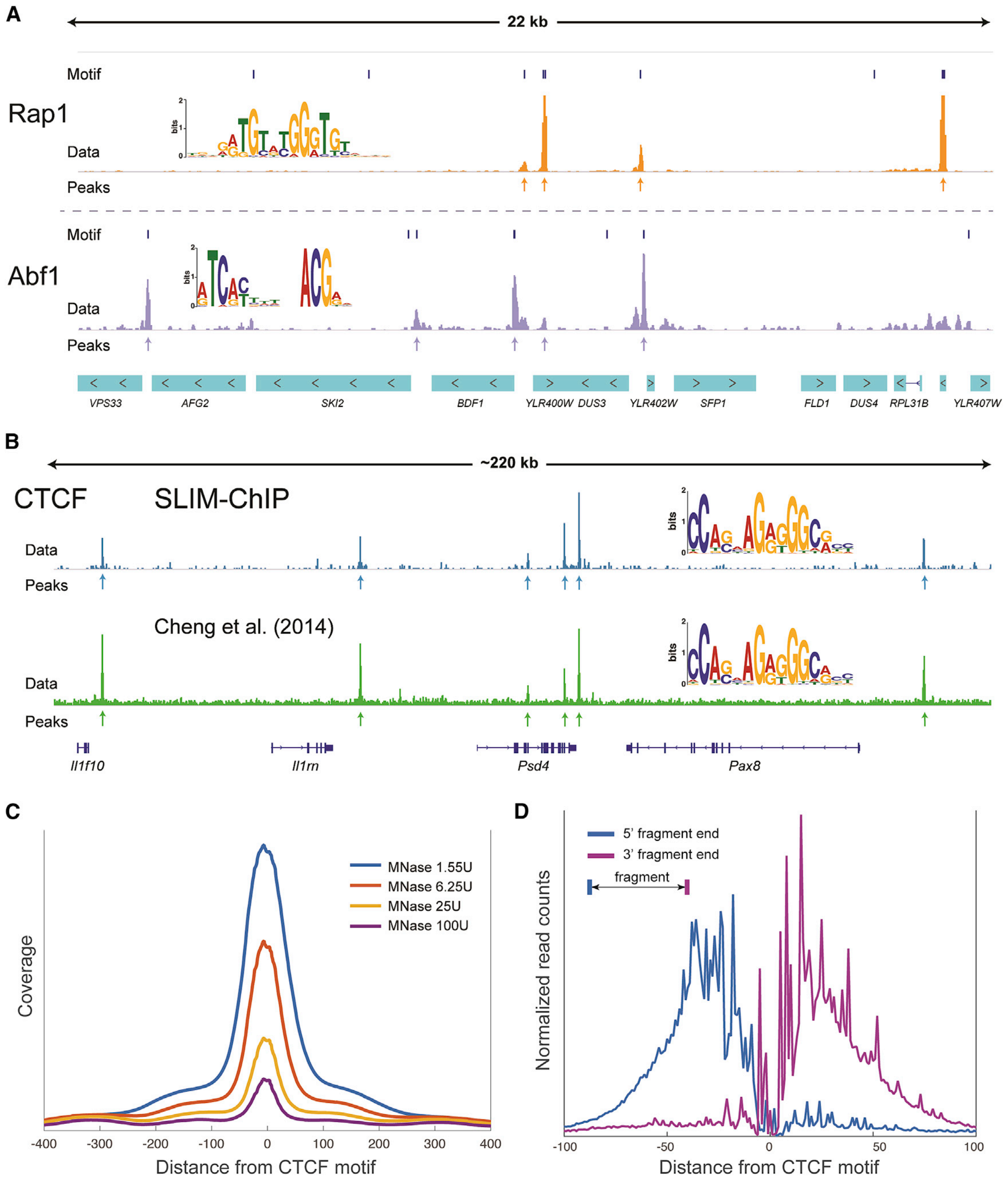

Figure 3. Profiling of Additional Yeast Transcription Factors and CTCF from mESCs by SLIM-ChIP

(A) Genome browser view of Abf1 and Rap1 binding. For each of the factors, a motif logo was generated from called peaks locations. See also Figure S3.

(B) Genome browser view comparing the CTCF-binding profile for SLIM-ChIP and Cheng et al. (2014). The motif logo for Cheng et al. was generated according to the peaks called in the original manuscript.

(C) The un-normalized coverage of CTCF ChIP signal around CTCF peaks is shown.

(D) The protection landscape around CTCF-binding peaks (as in Figure 2G).

the coverage at each site is relatively sparse, we represented each site as fragment length histogram (Figure 4B, bottom panels) and used K-means clustering to identify four clusters of Reb1 binding sites (Figure 4C). Cluster 1 and 2 likely represent isolated Reb1 binding. Cluster 1 is mostly composed of short fragments, while cluster 2 consists of a symmetric (relative to the TSS) continuum of fragment lengths that is sensitive to MNase levels (Figure S4B).

Binding sites at clusters 3 and 4 are characterized by bimodal distribution of fragment lengths in which the long fragments likely

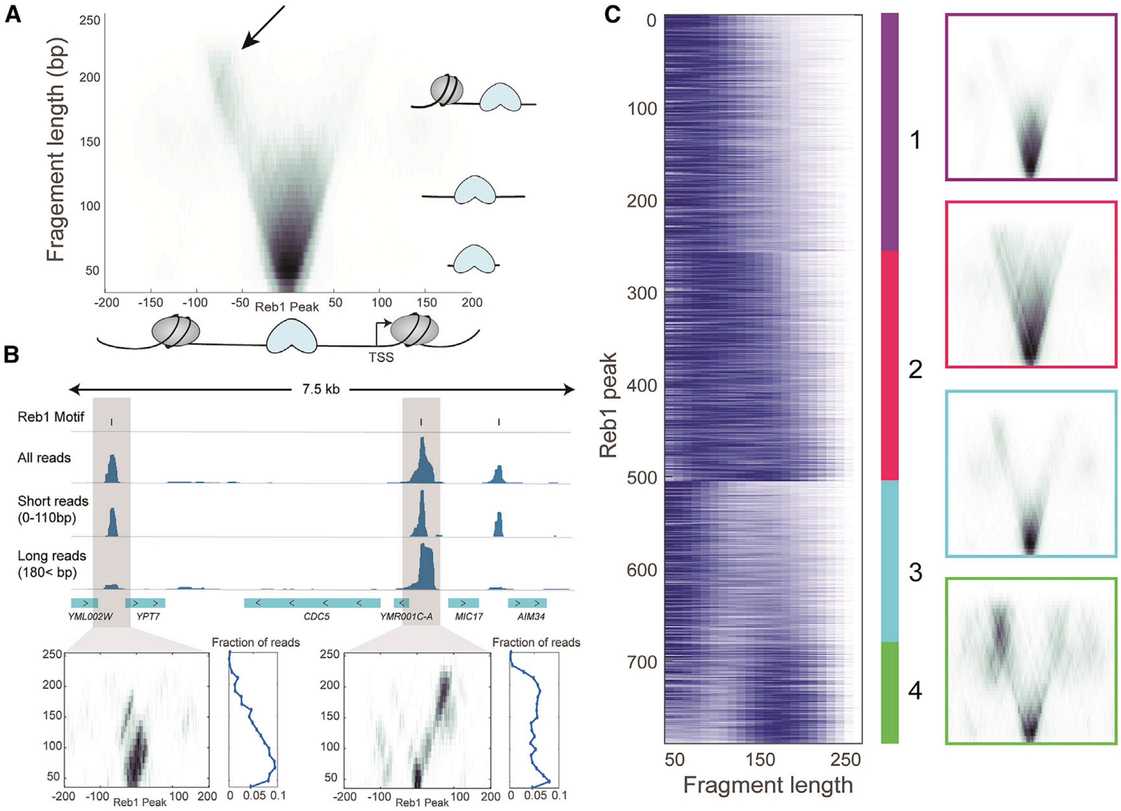

Figure 4. Reb1-Nucleosome Interactions at Bound Promoters

(A) Average V-plot of Reb1-enriched fragments aligned according to the nearest TSS. Each fragment is mapped to the x = middle of fragment, y = fragment length; darker areas indicate higher fragment density. Arrow points to fragments of length ~200 that cover the Reb1 site and a nucleosome-sized region, specifically the −1 nucleosome location.

(B) Example of two individual peaks with strikingly different V-plot (below) and the corresponding profile of fragment lengths. The peak on the right shows protection of intermediate length (< 100 bp) fragments, while the peak on the left shows two patterns, one very short (~50 bp) and the other long (~200 bp).

(C) Clustering of ~800 strong Reb1 peaks according to their fragment length profiles. We find four large clusters with different V-plots (as in A). Clusters 1–2 show protection of the Reb1-centered area. Clusters 3–4 show also protection of a flanking nucleosome. Cluster 4 shows clear preference to the −1 nucleosome. The cluster 4 pattern suggests that Reb1 interacts with the −1 nucleosome, and thus we observe protection of the long fragment covering both.

See also Figure S4.

represent an interaction of Reb1 with a proximal nucleosome. This is more apparent in cluster 4, which is heavily skewed toward long fragments (nucleosome interaction) and insensitive to MNase levels (Figure S4B). To further verify that the long fragments in cluster 4 represent Reb1-nucleosome interactions, we performed SLIM-ChIP with a general H3 antibody and generated V-plots mapping H3 binding around Reb1 sites at each cluster (Figure S4C). Indeed, part of the reads overlapping the −1 nucleosome in cluster 4 (but not in clusters 1 and 2) are shifted toward the Reb1-binding site and tend to be of longer length than −1 nucleosomes in clusters 1 and 2. Altogether, our results support the notion that longer fragments in cluster 4 represent tight interaction between Reb1 and the proximal −1 nucleosome.

Reb1-Interacting Nucleosomes Are Resistant to Remodeling by RSC

Reb1 promotes NDR formation partially by recruiting the essential RSC chromatin remodeling complex, which can evict or slide nucleosomes away from Reb1-binding sites (Hartley and Madhani, 2009; Krietenstein et al., 2016). We used an auxin degradation system (Morawska and Ulrich, 2013; Nishimura et al., 2009) to inducibly knock down Sth1, the catalytic subunit of RSC. MNase-seq following Sth1 depletion shows an expected movement of the Reb1-adjacent nucleosomes toward Reb1 (Figure S5A), consistent with the role of RSC in generating functional NDRs. We next examined the effect of depletion of Sth1 on nucleosomes from clusters 1 and 4. As expected, the +1 and −1 nucleosomes from cluster 1 move toward Reb1 upon Sth1 depletion (Figure 5A). In contrast, cluster 4 nucleosomes behave asymmetrically such that the +1 TSS-proximal nucleosome moves toward Reb1 while the Reb1-interacting nucleosome (−1) is much less affected (Figure 5A). This suggests that RSC is recruited to cluster 4 sites, and yet Reb1-interacting nucleosomes are unaffected by RSC activity. To test the hypothesis that remodeling by RSC prevents Reb1-nucleosome interactions, we performed SLIM-ChIP for Reb1 following depletion of Sth1. Indeed, we detect an increased proportion of long

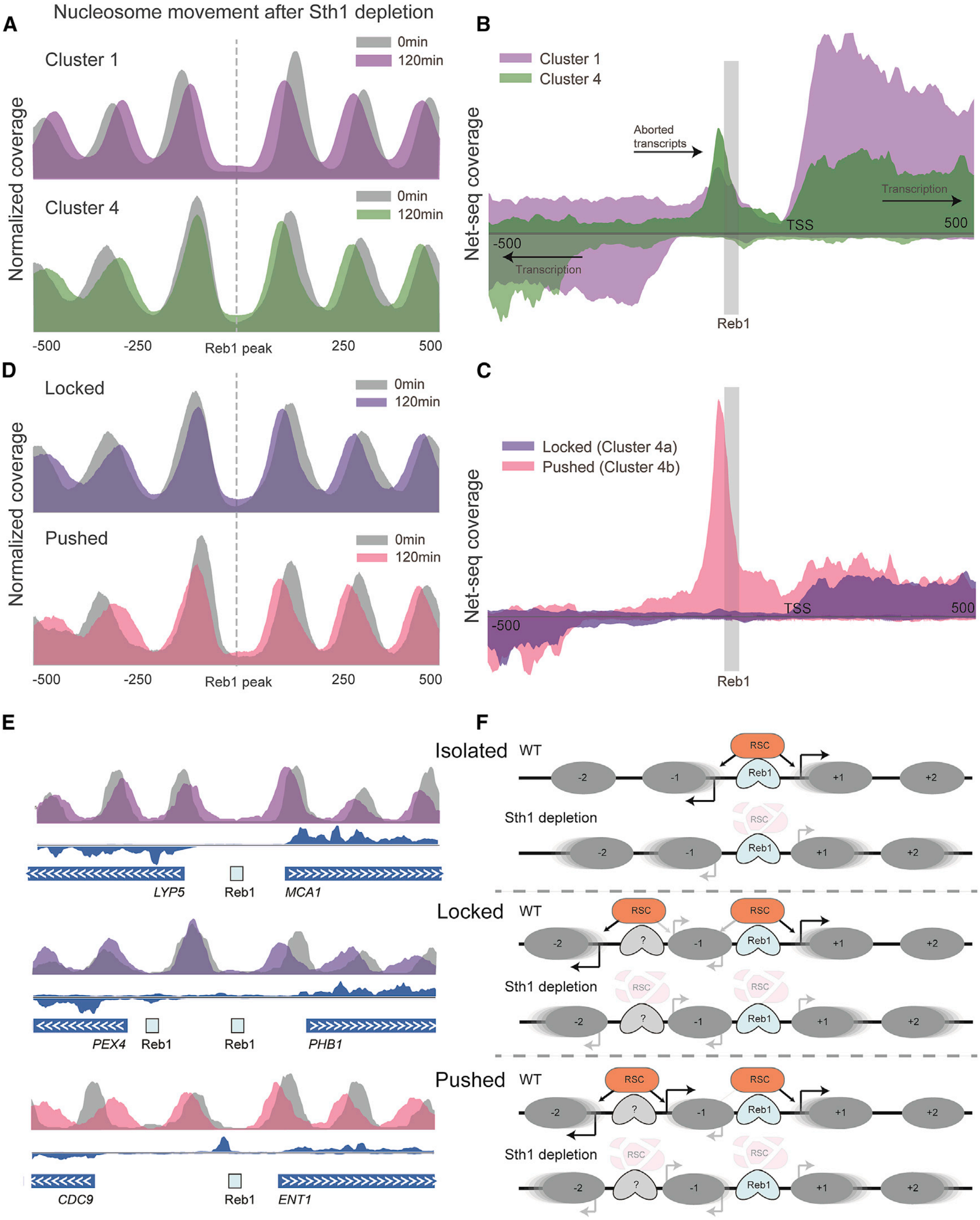

Figure 5. Two Mechanisms for Reb1-Nucleosome Interactions Establish Promoter Unidirectionality

(A) We used auxin-induced degradation to examine how promoter organization changes following depletion of Sth1 (the catalytic unit of the RSC complex). Showing average nucleosome occupancy before auxin addition (Sth1 is intact) and 120 min after addition (Sth1 is depleted for >90 min). Cluster 1 promoters exhibit the expected shift of nucleosomes −1 and +1 into the nucleosome-depleted region. Cluster 4 promoters show stable nucleosome −1, which is consistent with its co-occurrence with the bound Reb1 as suggested by the V-plot.

(B) Average transcribing Pol II occupancy (NET-seq) for clusters 1 and 4 aligned by the Reb1 site and oriented according to the nearest TSS. Top: transcription in the orientation of the TSS. Bottom: anti-sense transcription to the TSS. Both clusters show transcription start at the downstream TSS, but differ in the distance to anti-sense TSS. Cluster 4 also shows aborted short upstream transcripts terminating at the Reb1 site.

(C) Similar to (B) for two subclusters of cluster 4 divided according to presence of aborted upstream transcript (Figure S5D).

(legend continued on next page)

DNA fragments (Reb1 + nucleosome) around Reb1 sites upon Sth1 depletion in cluster 1, but not in cluster 4 (Figure S5B). This was specific for RSC, as depletion of other remodelers did not result in a similar effect.

Reb1-Nucleosome Interactions Affect Transcriptional Initiation

Reb1 is known to activate transcription (Brandl and Struhl, 1990; Kulkens et al., 1992), likely by establishing NDRs through DNA binding and subsequent recruitment of the RSC complex. We wondered whether stable Reb1-nucleosome interaction have functional consequences in terms of transcriptional activation and termination. We tested the profile of transcribing RNA polymerase II (Pol II) (NET-seq; Churchman and Weissman, 2011) around Reb1-binding sites (Figure S5C). Overall, transcription initiates bidirectionally around Reb1, albeit with higher signal in the direction of the nearest annotated TSS. This is in agreement with the bidirectional nature of promoters (Jin et al., 2017). We next focused on Reb1 binding sites in clusters 1 and 4 (Figure 5B). In cluster 1, transcription is again bidirectional such that it initiates symmetrically from both sides of Reb1 regardless of the location of the nearest annotated TSS (Figure 5B). This is in striking contrast to cluster 4, where we observe unidirectional transcription initiation almost exclusively from the nearest annotated TSS. It is likely that the inability of RSC to remodel the −1 nucleosome in cluster 4 is incompatible with transcription initiation. However, we do observe transcription initiation further upstream of the −1 nucleosome (−350 to the Reb1-binding site) due to an additional upstream NDR.

Two Mechanisms for Establishing Promoter Unidirectionality at Cluster 4 Sites

NET-seq patterns also reveal pronounced short aborted transcripts immediately adjacent to Reb1 at Cluster 4 sites (Figure 5B, arrow). Examining the patterns of transcription in the vicinity of cluster 4 sites, we observe two subpopulations, distinguished by presence of these aborted transcripts (Figures S5D and 5C). This suggests two separate local chromatin organizations around cluster 4 sites. Specifically, we hypothesize that chromatin organization in cluster 4a sites is incompatible with upstream transcriptional initiation, while organization in cluster 4b sites is compatible with such initiation.

The intimate connection between Reb1 NDR formation and transcription hinted that these differences in chromatin organization might affect the potential for RSC activity. Indeed, we observe that −1 nucleosomes in cluster 4a sites are locked in place and insensitive to Sth1 depletion. In contrast, −1 nucleosomes in cluster 4b sites slide away from Reb1 upon Sth1 depletion, suggesting that normally they are pushed against Reb1 by RSC (Figure 5D). Thus, cluster 4a sites have a nucleosome "locked" with Reb1, while cluster 4b sites have nucleosomes "pushed" against Reb1. We surmise that the insensitivity of −1 nucleosome to RSC depletion at cluster 4a (*locked*) sites is due to physical constraints, such as DNA-binding proteins, that limit the ability of the nucleosome to move away from the Reb1 site (Figure 5E, locked). As a consequence, the region upstream of the −1 nucleosome cannot accommodate RNA polymerase preinitiation complex (PIC) assembly. In contrast, in cluster 4b (pushed) sites, the same location is depleted of nucleosomes due to RSC activity, thus accommodating transcription initiation (Figure 5E, pushed). These observations suggest a model of RSC activity and transcription depending on chromatin organization (Figure 5F)

DISCUSSION

Here, we describe SLIM-ChIP, a straightforward, low input, high-resolution, automation-compatible ChIP method for mapping transcription factor binding. We show that it is robust to a range of MNase digestion levels and tolerant to low-input material. Mapping of benchmark transcription factors (Reb1 in yeast and CTCF in mammals) shows that SLIM-ChIP is in excellent agreement with previous results and yet it is much simpler to perform.

The combination of MNase digestion and recovery of variable fragment lengths allows us to gain important insights about binding events. Effectively, by combining MNase footprinting (Henikoff et al., 2011) with SLIM-ChIP, we can describe the DNA occupancy landscape in the subpopulations of cells where the factor is bound at a specific site. In particular, we show that there is a distinction between promoters where Reb1 is often co-bound with the adjacent −1 nucleosome on the same DNA molecule and promoters where such co-binding is not observed. As we show, this distinction functionally correlates both with the choice of TSSs and with RSC activity. In particular, the Reb1-adjacent nucleosome is insensitive to RSC activity and as such can interfere with NDR formation and transcription initiation. Further examination of these promoters suggests that Reb1-nucleosome co-binding can be achieved by at least two mechanisms: passive nucleosome "locking" or active nucleosome "pushing" (Figure 5F). Our findings for Reb1 suggest that transcription factor-nucleosome co-binding provides an epigenetic, potentially regulatable mechanism for blocking bidirectional transcription initiation events (Figure 5F).

Altogether, SLIM-ChIP is an attractive solution for mapping DNA-binding events that allows us to examine and interrogate the DNA occupancy landscape at a single-molecule level in the context of the binding event. This has the potential to elucidate mechanisms of transcriptional regulation that are not observed in the absence of informative fragment length data.

(D) Similar to (A) for the two subclusters, showing that the −1 nucleosome in the first subcluster is insensitive to RSC (locked), and in the other subcluster, the −1 is pushed toward Reb1 site by RSC.

(E) Examples of three sites belonging to cluster 1 (isolated), cluster 4a (locked), and cluster 4b (pushed), showing nucleosomes as in (A) and (D) and transcription as in (B) and (C).

(F) Schematic model of how Reb1 binding and RSC activity affect transcription in different promoter architectures.

See also Figure S5.

EXPERIMENTAL PROCEDURES

See Supplemental Experimental Procedures for detailed experimental steps.

Strains and Growth Conditions

All FLAG-tagged transcription factors strains were done in BY4741 background by inserting a 5xGly-FLAG His3MX6 cassette immediately before the target gene stop codon. In auxin depletion strains, an auxin-inducible degradation domain was genomically inserted immediately before the target gene stop codon in 2721 cells (Morawska and Ulrich, 2013). In all experiments, yeast cells were grown in YPD media at 30°C with constant shaking to optical density (OD) 0.6–0.8. When indicated, auxin (3-indolo acetic acid; Sigma) was added at a final concentration of 0.25 mM for the indicated time.

Mouse R1 ESCs (cell line) were grown on gelatin and maintained in ESC medium (DMEM, 15% ESC-grade fetal calf serum [FCS], 50 μg/mL penicillin, 50 μg/mL streptomycin, 2 mM L-glutamine, 1 mM sodium pyruvate, 0.1 mM nonessential amino acids, 0.1 mM β-mercaptoethanol, 1,000 U/ml leukemia inhibitory factor [LIF], PD0325901 inhibitor of MEK/ERK pathway [PD] 1 μM, and CHIR99021 inhibitor of GSK3 [CHIR] 3 μM).

Cell Fixation and MNase Digestion

Cells were fixed with 1% formaldehyde for 15 min. Yeast cells were treated with zymolyase to generate spheroplasts prior to lysis and MNase treatment. Lysed spheroplasts or mouse ESCs (mESCs) were treated with different amounts of MNase to digest chromatin into nucleosomes.

ChIP, Nucleosome Mapping, and DNA Sequencing

MNase-digested chromatin was allowed to bind to antibodies for 2–4 hr. Paramagnetic protein G dynabeads were added for an additional hour, and the beads were extensively washed. Bound chromatin was ligated to barcoded DNA adapters, and chromatin was reverse cross-linked and pooled. Barcoded chromatin was amplified with Illumina next-generation-sequencing compatible primers, and DNA libraries were paired-end sequenced by Illumina NextSeq 500. For MNase nucleosome mapping, MNase-digested chromatin was reverse cross-linked and isolated by 2× SPRI beads, and MNase sequencing libraries were prepared as described previously (Blecher-Gonen et al., 2013).

Sequence Analysis

Paired-end reads were mapped to the yeast (sacCer3) and mouse (mm9) genomes using bowtie2 default parameters, except for setting of maximal fragment size of 2,000 bp. Peak calling was performed using MACS algorithm (version 2.1.1) (Zhang et al., 2008). Motif discovery was done with the MEME-ChIP tool (Machanick and Bailey, 2011) using the default parameters.

Data Presentation

Meta-gene around the Transcription Factor Binding Motif

The location of the motif within the peak region was determined. The motif-containing regions were then oriented according to the motif directionality and aligned around its center. Peaks without a motif were discarded from this analysis.

Meta-gene around the TSS

The closest TSS to each peak summit was found. The genomic regions were then oriented according to the gene directionality and aligned around the TSS. Peaks that were >256 bp away from the nearest TSS were discarded from this analysis.

Finally, the sum of read coverage in the aligned regions was plotted.

Comparison of Reb1 Peaks

The peaks used for the comparison were reconstructed from the short reads (<80 bp) of the low-MNase sample (Figure 1B). The ChIP-exo and ORGANIC peak locations were downloaded from the original manuscripts (Kasinathan et al., 2014; Rhee and Pugh, 2011). In ChIP-exo, only the primary and monomer peaks were considered in the analysis. In ORGANIC, we used the peaks called using the "10′ MNase, 80mM NaCl" dataset. Peaks were considered identical in the overlap analysis if the distance between their centers was less than 64 bp.

Read Length Analysis

Peaks Clustering

Each peak was represented by a "fragment length vector" containing the fraction of reads mapped to the peak region in every length bin (40–250 bp, 10 bp resolution). These vectors were clustered using K-means algorithm (K = 4), generating 4 distinct clusters (Figure 4C). Peaks that did not contain the Reb1 motif (149 out of 1,078) and peaks with low read numbers (140 out of 929; bottom 15%) were discarded from this analysis.

NET-Seq Sequence Analysis

Raw NET-seq data was downloaded from the original manuscript (Churchman and Weissman, 2011). For NET-seq coverage plots, the 3′ read ends (marking the last bp incorporated by Pol II) were extended by 20 bp in the 5′ direction. Peaks that reside in telomeres were discarded from this analysis.

Sth1-Depletion Sequence Analysis

Nucleosome coverage plots were generated by taking the center of all mapped fragments and extending them by 25 bp in both directions. Peaks that reside in telomeres were discarded from this analysis.

DATA AND SOFTWARE AVAILABILITY

The accession number for the sequencing data reported in this paper is GEO: GSE108948.

SUPPLEMENTAL INFORMATION

Supplemental Information includes Supplemental Experimental Procedures and five figures and can be found with this article online at https://doi.org/10.1016/j.celrep.2018.02.052.

ACKNOWLEDGMENTS

We thank A. Appleboim, C. de Boer, S. Kadener, E. Meshorer, N. Moriel, O.J. Rando, K. Shekhar, H. Soreq, and members of the Friedman group for critical comments on the manuscript and figures. This work was supported by the ERC (grant 340712 to N.F.) and the Israeli Center of Research Excellence (I-CORE) on Chromatin and RNA in Gene Regulation (N.F. and O.R.).

AUTHOR CONTRIBUTIONS

Conceptualization, R.S., J.G., and N.F.; Methodology and Investigation, R.S. and J.G.; Software and Formal Analysis, J.G. and N.B.; Resources, D.J.-S. and A.K.-B. (Sth1 depletion data) and A.A. and O.R. (mESC); Writing and Visualization, N.F., J.G., and R.S.; Supervision and Funding Acquisition, N.F.

DECLARATION OF INTERESTS

The authors declare no competing interests.

Received: November 22, 2017
Revised: January 8, 2018
Accepted: February 12, 2018
Published: March 6, 2018

REFERENCES

Albert, I., Mavrich, T.N., Tomsho, L.P., Qi, J., Zanton, S.J., Schuster, S.C., and Pugh, B.F. (2007). Translational and rotational settings of H2A.Z nucleosomes across the Saccharomyces cerevisiae genome. Nature *446*, 572–576.

Angermayr, M., Oechsner, U., and Bandlow, W. (2003). Reb1p-dependent DNA bending effects nucleosome positioning and constitutive transcription at the yeast profilin promoter. J. Biol. Chem. *278*, 17918–17926.

Blecher-Gonen, R., Barnett-Itzhaki, Z., Jaitin, D., Amann-Zalcenstein, D., Lara-Astiaso, D., and Amit, I. (2013). High-throughput chromatin immunoprecipitation for genome-wide mapping of in vivo protein-DNA interactions and epigenomic states. Nat. Protoc. *8*, 539–554.

Brandl, C.J., and Struhl, K. (1990). A nucleosome-positioning sequence is required for GCN4 to activate transcription in the absence of a TATA element. Mol. Cell. Biol. *10*, 4256–4265.

Buenrostro, J.D., Giresi, P.G., Zaba, L.C., Chang, H.Y., and Greenleaf, W.J. (2013). Transposition of native chromatin for fast and sensitive epigenomic profiling of open chromatin, DNA-binding proteins and nucleosome position. Nat. Methods *10*, 1213–1218.

Cheng, Y., Ma, Z., Kim, B.-H., Wu, W., Cayting, P., Boyle, A.P., Sundaram, V., Xing, X., Dogan, N., Li, J., et al.; mouse ENCODE Consortium (2014). Principles of regulatory information conservation between mouse and human. Nature *515*, 371–375.

Churchman, L.S., and Weissman, J.S. (2011). Nascent transcript sequencing visualizes transcription at nucleotide resolution. Nature *469*, 368–373.

Colin, J., Candelli, T., Porrua, O., Boulay, J., Zhu, C., Lacroute, F., Steinmetz, L.M., and Libri, D. (2014). Roadblock termination by reb1p restricts cryptic and readthrough transcription. Mol. Cell *56*, 667–680.

Garber, M., Yosef, N., Goren, A., Raychowdhury, R., Thielke, A., Guttman, M., Robinson, J., Minie, B., Chevrier, N., Itzhaki, Z., et al. (2012). A high-throughput chromatin immunoprecipitation approach reveals principles of dynamic gene regulation in mammals. Mol. Cell *47*, 810–822.

Harbison, C.T., Gordon, D.B., Lee, T.I., Rinaldi, N.J., Macisaac, K.D., Danford, T.W., Hannett, N.M., Tagne, J.-B., Reynolds, D.B., Yoo, J., et al. (2004). Transcriptional regulatory code of a eukaryotic genome. Nature *431*, 99–104.

Hartley, P.D., and Madhani, H.D. (2009). Mechanisms that specify promoter nucleosome location and identity. Cell *137*, 445–458.

Henikoff, J.G., Belsky, J.A., Krassovsky, K., MacAlpine, D.M., and Henikoff, S. (2011). Epigenome characterization at single base-pair resolution. Proc. Natl. Acad. Sci. USA *108*, 18318–18323.

Jin, Y., Eser, U., Struhl, K., and Churchman, L.S. (2017). The ground state and evolution of promoter region directionality. Cell *170*, 889–898.e10.

Kasinathan, S., Orsi, G.A., Zentner, G.E., Ahmad, K., and Henikoff, S. (2014). High-resolution mapping of transcription factor binding sites on native chromatin. Nat. Methods *11*, 203–209.

Kent, N.A., Adams, S., Moorhouse, A., and Paszkiewicz, K. (2011). Chromatin particle spectrum analysis: a method for comparative chromatin structure analysis using paired-end mode next-generation DNA sequencing. Nucleic Acids Res. *39*, e26.

Koerber, R.T., Rhee, H.S., Jiang, C., and Pugh, B.F. (2009). Interaction of transcriptional regulators with specific nucleosomes across the Saccharomyces genome. Mol. Cell *35*, 889–902.

Krietenstein, N., Wal, M., Watanabe, S., Park, B., Peterson, C.L., Pugh, B.F., and Korber, P. (2016). Genomic Nucleosome Organization Reconstituted with Pure Proteins. Cell *167*, 709–721.e12.

Kulkens, T., van der Sande, C.A., Dekker, A.F., van Heerikhuizen, H., and Planta, R.J. (1992). A system to study transcription by yeast RNA polymerase I within the chromosomal context: functional analysis of the ribosomal DNA enhancer and the RBP1/REB1 binding sites. EMBO J. *11*, 4665–4674.

Lara-Astiaso, D., Weiner, A., Lorenzo-Vivas, E., Zaretsky, I., Jaitin, D.A., David, E., Keren-Shaul, H., Mildner, A., Winter, D., Jung, S., et al. (2014). Immunogenetics. Chromatin state dynamics during blood formation. Science *345*, 943–949.

Liaw, P.C., and Brandl, C.J. (1994). Defining the sequence specificity of the Saccharomyces cerevisiae DNA binding protein REB1p by selecting binding sites from random-sequence oligonucleotides. Yeast *10*, 771–787.

Liu, C.L., Kaplan, T., Kim, M., Buratowski, S., Schreiber, S.L., Friedman, N., and Rando, O.J. (2005). Single-nucleosome mapping of histone modifications in S. cerevisiae. PLoS Biol. *3*, e328.

Machanick, P., and Bailey, T.L. (2011). MEME-ChIP: motif analysis of large DNA datasets. Bioinformatics *27*, 1696–1697.

Mikkelsen, T.S., Ku, M., Jaffe, D.B., Issac, B., Lieberman, E., Giannoukos, G., Alvarez, P., Brockman, W., Kim, T.-K., Koche, R.P., et al. (2007). Genome-wide maps of chromatin state in pluripotent and lineage-committed cells. Nature *448*, 553–560.

Morawska, M., and Ulrich, H.D. (2013). An expanded tool kit for the auxin-inducible degron system in budding yeast. Yeast *30*, 341–351.

Nakahashi, H., Kieffer Kwon, K.-R., Resch, W., Vian, L., Dose, M., Stavreva, D., Hakim, O., Pruett, N., Nelson, S., Yamane, A., et al. (2013). A genome-wide map of CTCF multivalency redefines the CTCF code. Cell Rep. *3*, 1678–1689.

Nishimura, K., Fukagawa, T., Takisawa, H., Kakimoto, T., and Kanemaki, M. (2009). An auxin-based degron system for the rapid depletion of proteins in nonplant cells. Nat. Methods *6*, 917–922.

Rhee, H.S., and Pugh, B.F. (2011). Comprehensive genome-wide protein-DNA interactions detected at single-nucleotide resolution. Cell *147*, 1408–1419.

Sadeh, R., Launer-Wachs, R., Wandel, H., Rahat, A., and Friedman, N. (2016). Elucidating combinatorial chromatin states at single-nucleosome resolution. Mol. Cell *63*, 1080–1088.

Skene, P.J., and Henikoff, S. (2015). A simple method for generating high-resolution maps of genome-wide protein binding. eLife *4*, e09225.

Solomon, M.J., Larsen, P.L., and Varshavsky, A. (1988). Mapping protein-DNA interactions in vivo with formaldehyde: evidence that histone H4 is retained on a highly transcribed gene. Cell *53*, 937–947.

Teytelman, L., Thurtle, D.M., Rine, J., and van Oudenaarden, A. (2013). Highly expressed loci are vulnerable to misleading ChIP localization of multiple unrelated proteins. Proc. Natl. Acad. Sci. USA *110*, 18602–18607.

Tsankov, A.M., Gu, H., Akopian, V., Ziller, M.J., Donaghey, J., Amit, I., Gnirke, A., and Meissner, A. (2015). Transcription factor binding dynamics during human ES cell differentiation. Nature *518*, 344–349.

van Galen, P., Viny, A.D., Ram, O., Ryan, R.J.H., Cotton, M.J., Donohue, L., Sievers, C., Drier, Y., Liau, B.B., Gillespie, S.M., et al. (2016). A multiplexed system for quantitative comparisons of chromatin landscapes. Mol. Cell *61*, 170–180.

Vierstra, J., Wang, H., John, S., Sandstrom, R., and Stamatoyannopoulos, J.A. (2014). Coupling transcription factor occupancy to nucleosome architecture with DNase-FLASH. Nat. Methods *11*, 66–72.

Weiner, A., Hughes, A., Yassour, M., Rando, O.J., and Friedman, N. (2010). High-resolution nucleosome mapping reveals transcription-dependent promoter packaging. Genome Res. *20*, 90–100.

Weiner, A., Hsieh, T.-H.S., Appleboim, A., Chen, H.V., Rahat, A., Amit, I., Rando, O.J., and Friedman, N. (2015). High-resolution chromatin dynamics during a yeast stress response. Mol. Cell *58*, 371–386.

Weiner, A., Lara-Astiaso, D., Krupalnik, V., Gafni, O., David, E., Winter, D.R., Hanna, J.H., and Amit, I. (2016). Co-ChIP enables genome-wide mapping of histone mark co-occurrence at single-molecule resolution. Nat. Biotechnol. *34*, 953–961.

Zentner, G.E., Kasinathan, S., Xin, B., Rohs, R., and Henikoff, S. (2015). ChEC-seq kinetics discriminates transcription factor binding sites by DNA sequence and shape in vivo. Nat. Commun. *6*, 8733.

Zhang, Y., Liu, T., Meyer, C.A., Eeckhoute, J., Johnson, D.S., Bernstein, B.E., Nusbaum, C., Myers, R.M., Brown, M., Li, W., and Liu, X.S. (2008). Model-based analysis of ChIP-Seq (MACS). Genome Biol. *9*, R137.

Cell Reports, Volume *22*

Supplemental Information

Fine-Resolution Mapping of TF

Binding and Chromatin Interactions

Jenia Gutin, Ronen Sadeh, Nitzan Bodenheimer, Daphna Joseph-Strauss, Avital Klein-Brill, Adi Alajem, Oren Ram, and Nir Friedman

Supplementary Text: Detailed Protocol

Yeast growth

- Yeast cells were grown in YPD media at 30ºC with constant shaking to OD 0.6-0.8.

Yeast Cells fixation

- Add formaldehyde (1% final concentration) directly to cells and rotate 15 minutes at RT.
- Add glycine (0.125M final concentration from 2.5M stock) to the fixed cells and rotate 5 minutes at RT.
- Pellet cells by centrifugation (4000g, 5 minutes, 4ºC).
- Resuspend the cells pellet in ice cold ddH2O supplemented with a EDTA-free protease inhibitors cocktail (Roche).
- Pellet cells by centrifugation (4000g, 5 minutes, 4ºC).

Note: It is possible at this point to flash freeze the cell pellet and store at -80ºC.

Spheroplasting

- Resuspend the cell pellet in buffer Z (~ 5ul buffer Z per 1 OD600 of cells).
- Add zymolyase 20T (Seikagaku) at 0.15 - 0.5 units per 2x10^7 cells. Incubate cells at 30ºC for 20 minutes.
- To test spheroplasting efficiency, remove 1-5 µl of the cells into 1% SDS solution and check the cells under the microscope. Estimate spheroplasting efficacy (counted cells/expected # of cells*100). This number should be lower than 5%. If it is higher than 5% continue incubating the cells at 30ºC and test again.

MNase digestion (yeast)

Note: Since different TFs may exhibit distinct MNase sensitivity profiles, it is recommended to calibrate your MNase digest per specific TF by running a titration experiment. We find that 0.02-1 units of Worthington MNase per 10^7 cells to be a good range for initial titration. An alternative approach is to mix chromatin from different MNase digestion levels prior to immunoprecipitation.

- Pellet spheroplasts (6500 g, 10 minutes), remove the supernatant, and resuspend in NP buffer at final concentration of $0.2x10^7$ cells/µl.
- Prewarm samples to 37ºC for 5 minutes.
- Add MNase (Worthington) diluted in NP buffer for 20 minutes at 37ºC.
- Remove tubes to ice and add one volume of ice cold MNase stop buffer.

- Keep tubes on ice for 10-30 minutes (longer incubation increases yield), vortexed 3 x 10 seconds (this step increases chromatin yield but can be skipped when using 96 well plates to avoid sample spilling).
- Centrifuge samples (16,000g, 10 minutes, 4ºC - for 96 wells plates centrifuge 30 minutes at 5000g, 4ºC).
- Remove the supernatant containing the chromatin to fresh tubes or 96 well plate.

mESCs fixation and lysis: Cells were fixed with 1% formaldehyde for 15 minutes at room temperature, following by quenching with 0.125M glycine for 5 minutes at room temperature.

- Centrifuge cells, remove supernatant, and wash with ice cold PBS.
- Suspend cell pellet in 1 volume of ice cold PBS to get 10^7 cells/ml.
- Add equal volume of ice cold 2X cell lysis buffer
- Add the desired amount of MNase
- Mix well by pipetting up and down and incubate on ice for 10 minutes.
- Incubate 15 minutes at 37°C
- Stop MNase by adding 20mM EDTA.
- Incubate on ice for 30 minutes
- Centrifuged samples (16,000g, 10 minutes, 4ºC - for 96 wells plates centrifuge 30 minutes at 5000g, 4ºC).
- Remove the supernatant containing the chromatin to fresh tubes or 96 well plate.

MNase digest evaluation

- Remove 2-10µl of MNase digested chromatin to a fresh tube and adjust volume to 9ul with EB.
- Add 1µl of 0.5ug/µl RNase A and incubate for for 30 minutes at 37ºC.
- Add 40µl of proteinase K solution (10 mM Tris pH 8.0, 5 mM EDTA, 300 mM NaCl, 0.6% SDS) containing 50 units of proteinase K.
- Incubate for 2 hours at 37ºC, and for 12-16 hours at 65ºC.
- Isolate DNA by 2X SPRI beads cleanup, resuspend DNA in 20µl of 10mM Tris pH-8.0, and measure DNA concentration by Qubit.
- Expect total of ~ 10-100 ng of DNA
- Visualize DNA by TapeStation (Agilent) or agarose gel.

Chromatin immobilization

Note: The amount of DNA used for ChIP can vary and depends on the abundance of the target and antibody yield and specificity.

- Remove MNase digested chromatin samples to a fresh 96 well plate and adjust the volume to 80 µl with ice cold RIPA buffer and antibody (for specific details see antibodies section below).
- Incubate the samples with gentle tumbling for 2 hours to O/N at 4ºC.
- While samples are incubated wash protein G dynabeads three times in RIPA (yeast) or cell lysis buffer buffer (mESCs) (20µl beads per sample). Resuspend beads to the original volume with RIPA or cell lysis buffer.
- Centrifuge the samples shortly, add 20µl of protein G to each sample, and incubate the samples with gentle tumbling for an additional hour at 4ºC.
- Magnetize the samples and wash: 6 X RIPA buffer, 3 X RIPA 500 containing 500 mM NaCl), 3 X LiCl wash buffer. It is possible to use vacuum for these washes.
- **Important**: From this point on DO NOT use vacuum to aspirate the supernatant.
- Wash the beads 3 X 10 mM Tris pH 7.5 supplemented with protease inhibitors.

Chromatin barcoding

End repair:

- Resuspend the immobilized chromatin in 10µl of 10 mM Tris pH 7.5.
- Add 15 µl of end repair mixture and mix well by pipetting.
- Incubate for 22 minutes at 12°C followed by 22 minutes at 25°C.
- Magnetize beads and wash once in 150µl 10 mM Tris pH 8.0 and resuspend the beads in 20µl of 10 mM Tris pH 8.0.

A base addition:

- Add 10 µl of A-Base mix [10 mM Tris pH 8, 10 mM $MgCl_2$, 50 mM NaCl, 1 mM DTT, 0.58 mM dATP, 0.75 units Klenow fragment (NEB)] to the beads and mix well by pipetting.
- Incubate samples at 37°C for 30 minutes.
- Magnetize beads and wash once in 150µl 10 mM Tris pH 8.0 and resuspend the beads in 9µl of 10 mM Tris pH 8.0.

Adapters ligation:

- Add 2.5µl of indexed adapters (Blecher-Gonen et al., 2013) to each sample and mix well by pipetting. Add 17µl of ligation mix [14.5 µl of 2X quick ligase buffer (NEB), or Rapid ligation buffer (lucigen), and 2.5µl quick ligase (NEB) or NxGen T4 ligase], mix well by pipetting, and incubate at 25°C for 45 minutes.
- Magnetize beads and wash 3x 150µl RIPA

Note: Ligation of adaptors increases the size of DNA fragments bound by TFs and allow the isolation of fragments that are < 50bp (prior to adapter ligation) in subsequent steps.

Reverse crosslinks and DNA cleanup

- Resuspend the beads in 24µl of chromatin elution buffer supplemented with 1µl of 0.5µg/µl RNase A and incubate for for 30 minutes at 37ºC.
- Add 24µl of chromatin elution buffer supplemented with 1 µl of proteinase K (50 units/µl, epicenter).
- Incubate for 2 hours at 37ºC, and for 12-16 hours at 65ºC.
- At this point you can pool samples
- Isolate DNA by 2 X SPRI beads cleanup, resuspend DNA in 25µl of 10mM Tris pH-8.0.

Note: At this point it is possible to run a 4% agarose gel (E-Gel® EX Agarose Gels, 4%, Invitrogen) and gel purify DNA of desired size. This step can be skipped but it is recommended for elimination of adapter dimers and reduce background of nucleosomal DNA. We normally isolate DNA fragments of ~170-400bp which correspond to unligated fragments of 30-260bp.

Library amplification:

- Remove 23µl of the eluted chromatin into a fresh PCR tube. Add 2µl of barcoded amplification primers mix (Different barcode for each antibody used in second ChIP, see primer sequence below) and 25µl of 2 X Kapa hifi hotstart ready mix.
- Run PCR for 12-16 cycles.
  The number of PCR cycles depends on the ChIP yield. It is recommended to use the lowest number of cycles that yield sufficient library for next generation sequencing to reduce PCR duplicates.
- Isolate DNA by 0.8 X SPRI beads cleanup, resuspend in 20µl of 10mM Tris pH-8.0, and measure DNA concentration by Qubit.
  The total amount of amplified DNA can vary between 40-1000 ng of DNA.
- Visualize DNA by TapeStation. For example see Figure S1A.

Forward amplification primer:
5’ - AATGATACGGCGACCACCGAGATCTACAC [8bp barcode] ACACTCTTTCCCTACACGAC
Reverse amplification primer:
5’ - CAAGCAGAAGACGGCATACGAGAT

Buffers:

Buffer Z: 1 M sorbitol, 50 mM Tris 7.4, 10 mM β-mercaptoethanol (freshly added).

NP buffer: 10 mM Tris pH 7.4, 1 M sorbitol, 50 mM NaCl, 5 mM MgCl2, 1 mM CaCl2, and 0.075% NP-40, freshly supplemented with 1 mM β-mercaptoethanol, 500 µM spermidine, and EDTA-free protease inhibitor cocktail.

Cell lysis buffer: 50 mM Tris pH 8.0, 150 mM NaCl, 1% Triton X-100, 0.1% sodium deoxycholate, 5 mM CaCl2, EDTA-free protease inhibitor cocktail

MNase stop buffer: 220 mM NaCl, 0.2% SDS, 0.2% sodium deoxycholate, 10 mM EDTA, 2%,Triton X-100, EDTA-free protease inhibitor cocktail.

RIPA buffer: 10 mM Tris pH 8.0, 140 mM NaCl, 1 mM EDTA, 0.1% SDS, 0.1% sodium deoxycholate, 1% Triton X-100, EDTA-free protease inhibitor cocktail.

LiCl wash buffer: 10 mM Tris pH 8.0, 0.25 M LiCl, 0.5% NP-40, 0.5% Sodium Deoxycholate, 1 mM EDTA, EDTA-free protease inhibitor cocktail

End Repair mixture: 100 mM Tris pH 7.5, 20 mM MgCl2, 20 mM DTT, 2 mM ATP, 0.8 mM each dATP , dCTP, dGTP, dTTP, 12.5 units T4 polynucleotide kinase (NEB), 0.5 units T4 polymerase (NEB).

A-Base mix: 10 mM Tris pH 8, 10 mM MgCl2, 50 mM NaCl, 1 mM DTT, 0.58 mM dATP, 7.5 units Klenow fragment (NEB).

Chromatin elution buffer: 10 mM Tris pH 8.0, 5 mM EDTA, 300 mM NaCl, 0.6% SDS

Antibodies

The following antibodies were used in this study:

Antigen	Catlog #	µg antibody / ChIP
Anti Flag (M2)	Sigma	2
Anti CTCF	07-729 (Millipore)	2

Figure S1

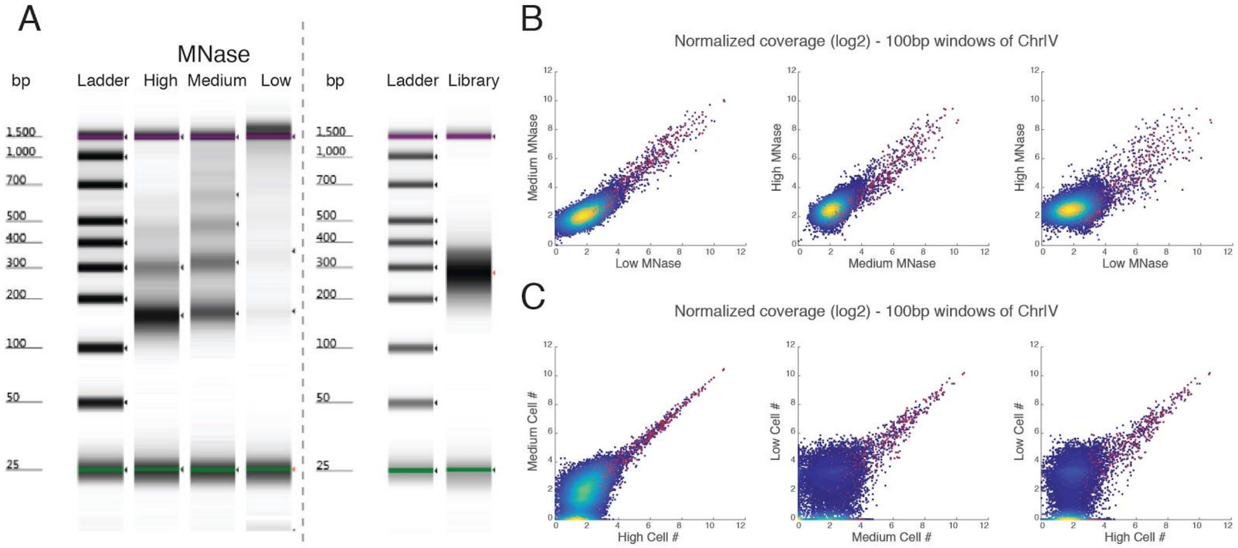

Figure S1: Comparison of MNase digestions and Input materials. Related to Figure 1B. **A.** Tape station chromatography of MNase digested DNA (left panel) or SLIM-ChIP library (right panel). Higher levels of MNase are characterized by larger proportion of mononucleosomes and shorter nucleosomal length. SLIM-ChIP library was isolated from gel as described in Methods. **B-C.** Scatter plots showing the sum of read coverage in 100bp windows across chrIV for different MNase concentrations and different cell numbers (as in Figure 1B). Locations of called peaks are highlighted.

Figure S2

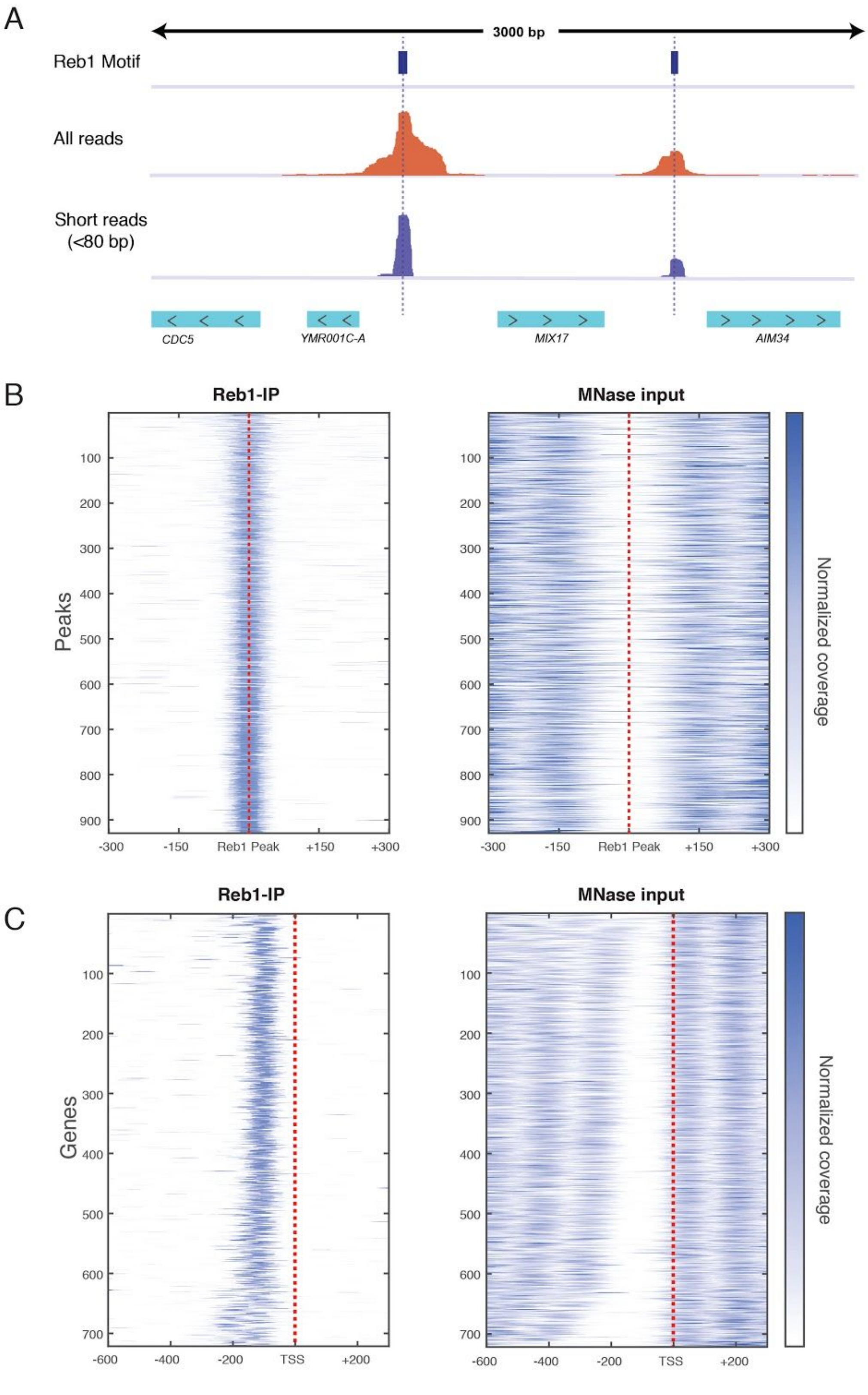
A
3000 bp
Reb1 Motif
All reads
Short reads
(<80 bp)
CDC5
YMR001C-A
MIX17
AIM34
B
Reb1-IP
MNase input
Peaks
100
200
300
400
500
600
700
800
900
-300
-150
Reb1 Peak
+150
+300
Normalized coverage
C
Reb1-IP
MNase input
Genes
100
200
300
400
500
600
700
-600
-400
-200
TSS
+200
Normalized coverage

Figure S2: Reb1 peak coverage. Related to Figures 1B-D. **A.** Genome browser view illustrating the signal from all reads versus short reads (<80bp). Focusing on short reads tightens the signal around the Reb1 binding motifs. **B.** Heat map of the read coverage of Reb1 ChIP signal (short reads) and MNase input signal(Weiner et al. 2015) centered on Reb1 peak (as in Figure 1C). **C.** Heat map of the same signals as B, aligned according to the TSS of genes with a Reb1 peak in their promoter.

Figure S3

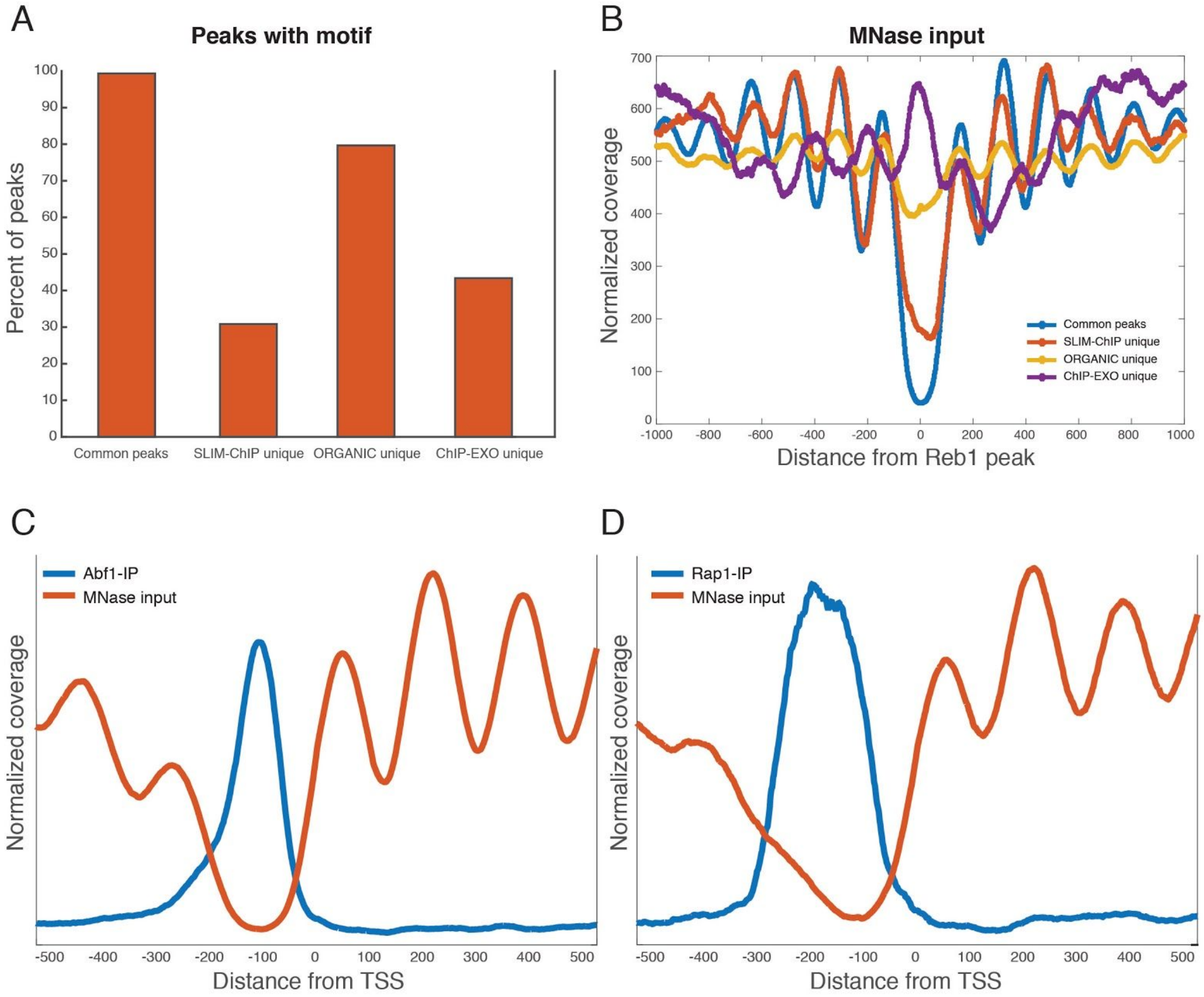

Figure S3: Reb1 across methods and profiling additional yeast transcription factors. **Related to Figure 2B and 3A.**. **A.** For selected groups in Figure 2B the percent of peaks with a motif is shown. The vast majority of common peaks contain a Reb1 binding motif. **B.** For selected groups in 2B the normalized coverage of MNase input (Weiner et al. 2015) signal around Reb1 peak centers is plotted. **C-D.** Normalized coverage of Abf1,Rap1 (short reads, 0-80bp) and MNase input signals around TSS. For each factor only promoters that contain a motif are used. MNase input track was adapted from Weiner et. al.

Figure S4

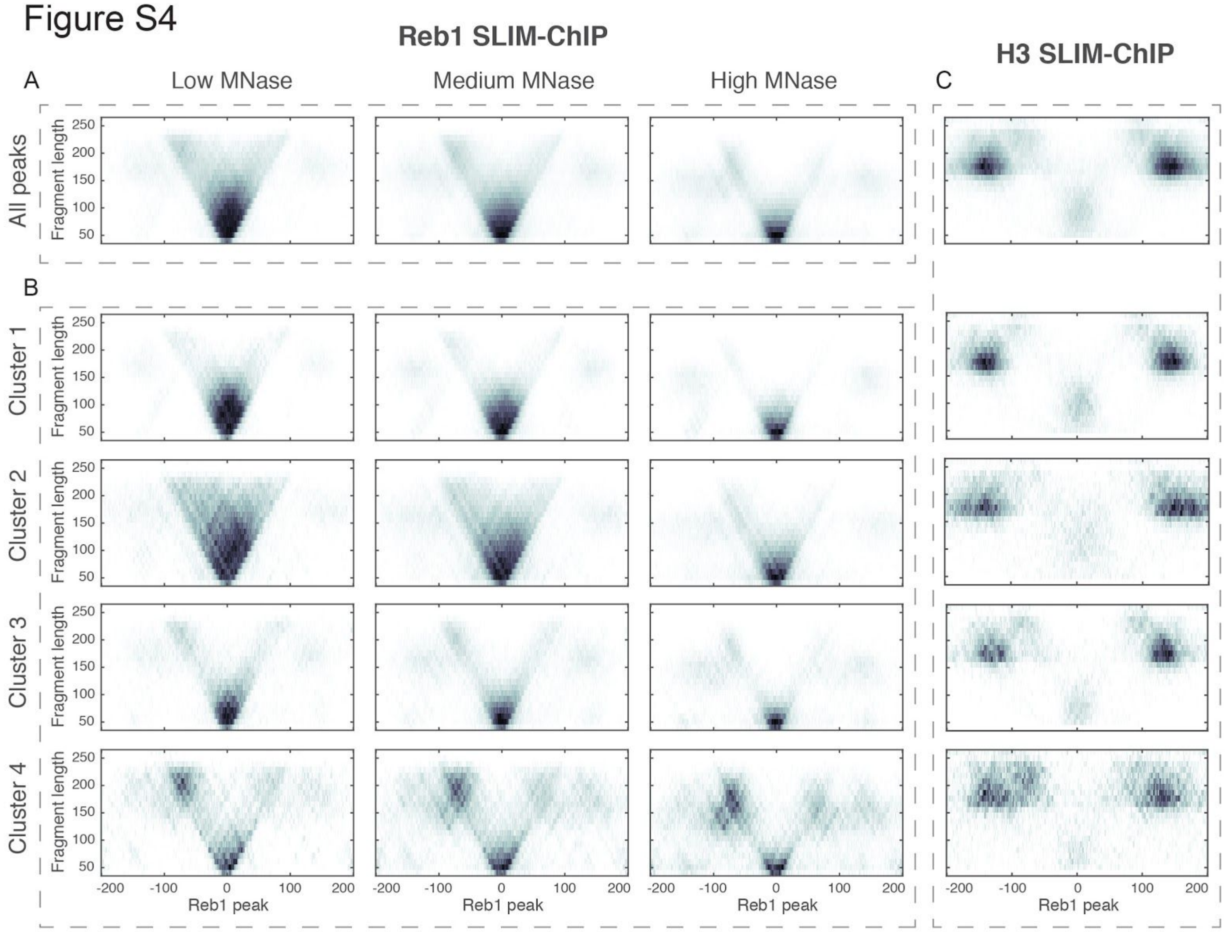

Figure S4: MNase sensitivity of read length patterns in clusters of Reb1 sites. Related to Figure 4. **A**. V-plots of all Reb1 sites in three MNase levels (as in Figure 4A). **B.** Same as A for each of the four clusters (as in Figure 4C). **C**. V-plots of H3 SLIM-ChIP for the groups shown in A and B.

Figure S5

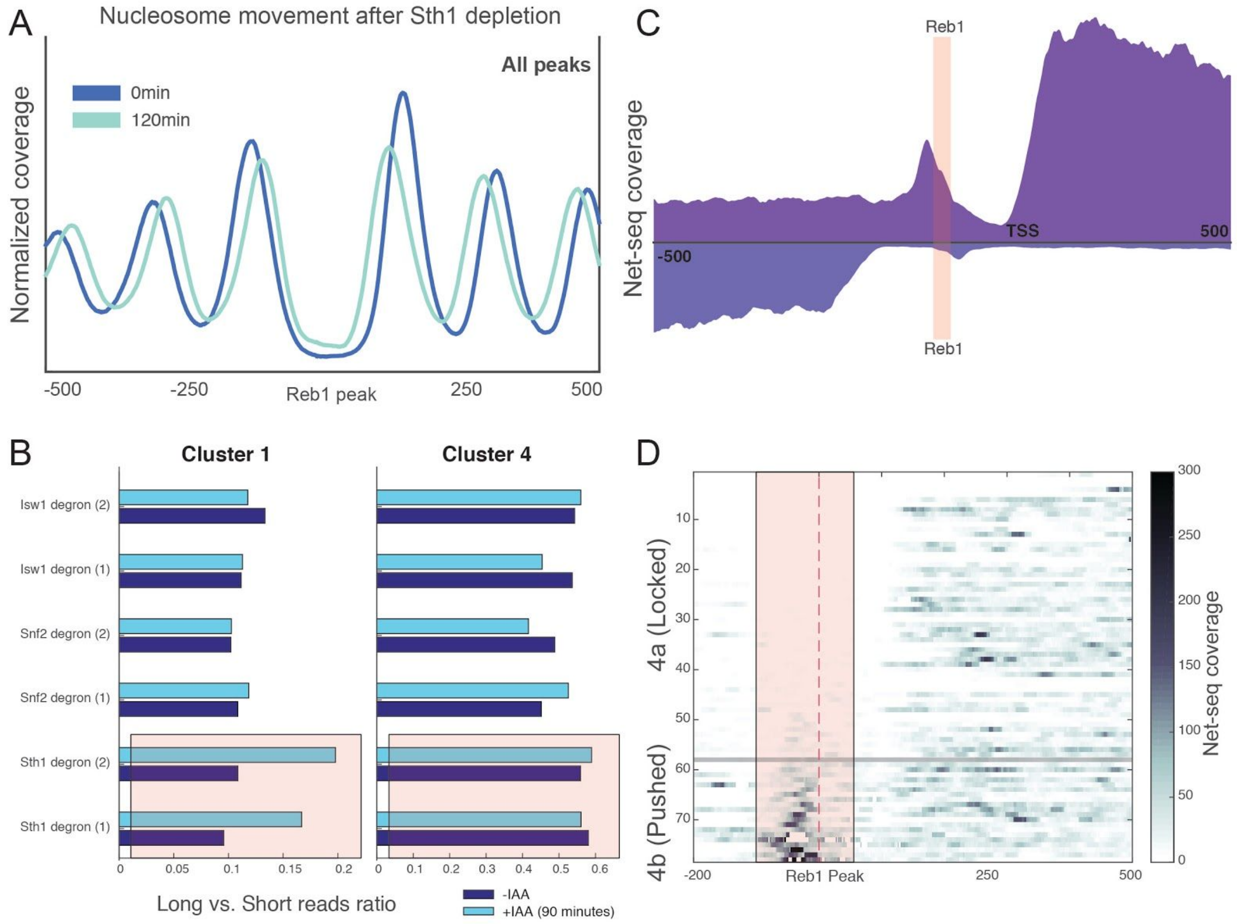

Figure S5: Effects of RSC depletion on Reb1 binding sites. Related to Figure 5. **A.** Nucleosome movement in Sth1 depletion for all Reb1 sites (as in Figure 5A,D). **B.** Examining change in fragment length in Reb1 SLIM-ChIP in three strains with depletion of a catalytic units of chromatin remodelers (Sth1 of RSC, Snf2 of SWI/SNF, Isw1 of ISW1a/b). Showing two repeats for each strain, comparing the ratio of total coverage of long fragment (>180bp) to the total coverage of short fragments (<110bp) counting over Reb1 sites in two clusters. Only in Sth1 depletion at Cluster 1 sites we observe a shift in the ratio. This shift corresponds to nucleosomes shifting to be adjacent to Reb1 when RSC is not active. **C.** Average NET-seq coverage at Reb1 binding sites (as in Figure 5B,C). **D.** Heat map of sense direction NET-seq coverage around each of the Reb1 sites in Cluster 4, oriented according to the nearest TSS. Rows (sites) are sorted according to total coverage in the highlighted region. We observe two subpopulations, one without transcription in the region and the other with.
